# Coordination of photosynthetic traits across soil and climate gradients

**DOI:** 10.1111/gcb.16501

**Published:** 2022-11-16

**Authors:** Andrea C. Westerband, Ian J. Wright, Vincent Maire, Jennifer Paillassa, Iain Colin Prentice, Owen K. Atkin, Keith J. Bloomfield, Lucas A. Cernusak, Ning Dong, Sean M. Gleason, Caio Guilherme Pereira, Hans Lambers, Michelle R. Leishman, Yadvinder Malhi, Rachael H. Nolan

**Affiliations:** ^1^ Faculty of Science and Engineering School of Natural Sciences Macquarie University North Ryde New South Wales Australia; ^2^ Hawkesbury Institute for the Environment Western Sydney University Penrith New South Wales Australia; ^3^ Département des Sciences de l'environnement Université du Québec à Trois‐Rivières Trois‐Rivières Québec Canada; ^4^ Georgina Mace Centre for the Living Planet Imperial College London Ascot UK; ^5^ Department of Earth System Science Tsinghua University Beijing China; ^6^ Australian Research Council Centre of Excellence in Plant Energy Biology Research School of Biology The Australian National University Canberra Australian Capital Territory Australia; ^7^ College of Science and Engineering James Cook University Cairns Queensland Australia; ^8^ USDA‐ARS Water Management and Systems Research Unit Fort Collins Colorado USA; ^9^ Department of Civil and Environmental Engineering Massachusetts Institute of Technology Cambridge Massachusetts USA; ^10^ School of Biological Sciences University of Western Australia Perth Western Australia Australia; ^11^ School of Geography and the Environment Environmental Change Institute University of Oxford Oxford UK

**Keywords:** Australia, least‐cost theory of photosynthesis, nutrient‐use efficiency, optimality theory, plant functional traits, soil nutrients, soil phosphorus, trait coordination, water‐use efficiency

## Abstract

“Least‐cost theory” posits that C_3_ plants should balance rates of photosynthetic water loss and carboxylation in relation to the relative acquisition and maintenance costs of resources required for these activities. Here we investigated the dependency of photosynthetic traits on climate and soil properties using a new Australia‐wide trait dataset spanning 528 species from 67 sites. We tested the hypotheses that plants on relatively cold or dry sites, or on relatively more fertile sites, would typically operate at greater CO_2_ drawdown (lower ratio of leaf internal to ambient CO_2_, *C*_i_:*C*_a_) during light‐saturated photosynthesis, and at higher leaf N per area (N_area_) and higher carboxylation capacity (*V*_cmax 25_) for a given rate of stomatal conductance to water vapour, *g*_sw_. These results would be indicative of plants having relatively higher water costs than nutrient costs. In general, our hypotheses were supported. Soil total phosphorus (P) concentration and (more weakly) soil pH exerted positive effects on the N_area_–*g*_sw_ and *V*_cmax 25_–*g*_sw_ slopes, and negative effects on *C*_i_:*C*_a_. The P effect strengthened when the effect of climate was removed via partial regression. We observed similar trends with increasing soil cation exchange capacity and clay content, which affect soil nutrient availability, and found that soil properties explained similar amounts of variation in the focal traits as climate did. Although climate typically explained more trait variation than soil did, together they explained up to 52% of variation in the slope relationships and soil properties explained up to 30% of the variation in individual traits. Soils influenced photosynthetic traits as well as their coordination. In particular, the influence of soil P likely reflects the Australia's geologically ancient low‐relief landscapes with highly leached soils. Least‐cost theory provides a valuable framework for understanding trade‐offs between resource costs and use in plants, including limiting soil nutrients.

## Graphical abstract

Using an Australia‐wide photosynthetic trait dataset, we tested predictions from least‐cost optimality theory, which posits that plants balance nutrient use and water loss to optimize photosynthesis. We found that leaf nitrogen and rates of water loss co‐vary with one another in response to climate and to a lesser extent, soils. While much attention has been paid to the role of climate in driving photosynthesis, fewer studies have investigated soil effects, despite the important role of soil in limiting plant growth and in shaping the distributions of vegetation communities.

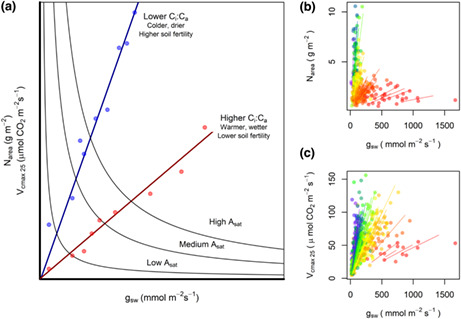

## 1 INTRODUCTION

Photosynthesis is a fundamental process in the global carbon cycle, governing flows of energy (Friend et al., 2009; Smith & Dukes, 2013). Broad‐scale influences of site climate on photosynthesis and associated traits have been widely reported. For example, leaf nitrogen concentration on an area basis (N_area_) and area‐based rates of light‐saturated photosynthesis, *A*_sat_, are typically higher on relatively arid sites (Wright et al., 2005). Central to determining rates of photosynthesis is the internal concentration of CO_2_ within leaves (*C*_i_), as the ratio of intercellular to atmospheric CO_2_ concentration (*C*_i_:*C*_a_) represents the balance between CO_2_ demand (from the photosynthetic carboxylating enzyme, Rubisco) and supply (via stomata) during photosynthesis. Typically, *C*_i_:*C*_a_ corresponding with *A*_sat_ shows clear patterning with a variety of climate variables, being generally lower at arid, high‐altitude and cold sites (Cornwell et al., 2018; Dong et al., 2020; Prentice et al., 2011, 2014). Carboxylation capacity (*V*_cmax_) considered at ambient temperatures tends to be higher at warmer sites (Dong et al., 2022) and, at least within‐species, is generally higher in summer than in winter (Bloomfield et al., 2018). Conversely, *V*_cmax_ normalized to a standard temperature (commonly 25°C) tends to be lower in summer than in winter (Bloomfield et al., 2018; Hikosaka et al., 2007; Lin et al., 2013) and declines with increasing growth temperature (Dong et al., 2017; Scafaro et al., 2017; Togashi et al., 2018). Finally, stomatal conductance to water, *g*_sw_, shows little patterning with site climate, at least at a global scale: individually or together, site temperature and precipitation explain <1% variation in *g*_sw_ in the global trait dataset of Wright, Reich, et al. (2004). Within C_3_ woody angiosperms measured across major terrestrial biomes, there is no relationship between *g*_sw_ and either mean annual temperature (MAT), photosynthetically active radiation (PAR), or atmospheric vapor pressure deficit (VPD) (Murray et al., 2019, 2020).

Broad‐scale influences of soil properties on photosynthetic traits are less well documented but this area of research is growing. Maire et al. (2015) found that *A*_sat_ increased with increasing soil pH and decreased weakly with increasing soil organic C concentration but had no relationship with soil N or available P concentration. N_area_ is higher on sites with high soil pH (Dong et al., 2020; Maire et al., 2015) and negatively correlated with soil organic C and soil total N concentrations, albeit weakly (Maire et al., 2015). Ordoñez et al. (2009) reported higher mass‐based nitrogen concentrations at sites with faster N‐mineralization rates (argued to be a more relevant index of plant‐available N than soil total N concentration) but found no relationship between N_area_ and N‐mineralization rate due to a concomitant increase in leaf area per unit mass (specific leaf area, SLA), where N_area_ is N_mass_ divided by SLA. Dong et al. (2020) reported lower *C*_i_:*C*_a_ on high pH soils, as did Cornwell et al. (2018) and Paillassa et al. (2020). Paillassa et al. (2020) explored the role of soil textural properties and reported higher *V*_cmax_ coupled with higher *g*_sw_ on sites with high soil silt content, lower *C*_i_:*C*_a_ on deeper soils, and higher *C*_i_:*C*_a_ in areas of high soil silt content, the last of which was also reported by Cornwell et al. (2018). *g*_sw_ is higher on soils with low plant‐available P concentration (Maire et al., 2015), although studies on soil P effects are scarce.

Soil pH, often described as a “master soil variable,” has emerged as an important explanatory variable in several studies of plant trait variation. Globally, soil pH tends to be higher at more arid than at mesic sites (Slessarev et al., 2016), although in Australia acid soils also occur at arid sites, likely owing to its low‐relief landscape and the predominance of highly leached, ancient soils (Kooyman et al., 2017). Previous studies have worked to decouple the effects of pH and aridity. Presumably, the effect of pH on photosynthetic traits relates to its influence on soil nutrient availability: broadly speaking, nutrient availabilities are highest at mid‐range pH values and lowest on extremely alkaline or acid soils. This can occur via changes in solubility and oxidation states (Lambers & Oliveira, 2019), enzymatic activity (Sinsabaugh et al., 2008; Sinsabaugh & Follstad Shah, 2012) and shifts in the activity and diversity of soil micro‐organisms involved in nutrient cycling (Fierer & Jackson, 2006; Lauber et al., 2008). Hence, N and P availability are generally highest at intermediate levels of soil pH, driving shifts in key plant functions, including photosynthesis.

“Least‐cost” theory (Wright et al., 2003) is a framework for understanding the coordination of water and nutrient use during photosynthesis, and how it varies with site climate and soil properties. Under this theory, photosynthesis is conceptualized as a production process with two key inputs, N and water, which are associated with *V*_cmax_ and the transpiration pathway, respectively. Based on standard microeconomic theory for a two‐factor production process, the optimal balance of these inputs—indicating the lowest total cost for a given level of production—is set by the ratio of the *unit costs* of the two resources. A key concept of the theory is *substitutability*: in principle, plants can economize on water use by “spending” more on leaf N (i.e., all else being equal, higher N_area_ at a given *g*_sw_ results in higher *V*_cmax_ and hence lower *C*_i_:*C*_a_), or economize on N use by operating at a higher *g*_sw_ or transpiration rate. Thus, the approach integrates the single‐resource concepts of photosynthetic nitrogen‐use efficiency and water‐use efficiency (Field et al., 1983; Lambers & Oliveira, 2019; Smith et al., 1997).

Briefly summarizing, the following are key assumptions from least‐cost theory (Prentice et al., 2014; Wang et al., 2017; Wright et al., 2003): (1) the unit cost for carboxylation or N_area_ is set by the combined costs of acquiring soil nutrients needed for photosynthetic enzymes and the respiratory costs of building and maintaining enzyme function (e.g., protein turnover); (2) soil nutrients are more expensive to acquire when at lower availability (e.g., from higher root construction costs; more carbon traded for nutrients with mycorrhizas; higher costs associated with producing root exudates, such as carboxylates or phosphatases); (3) the unit cost for transpiration is set by the cost of acquiring soil water and the respiratory costs of maintaining functional sapwood; (4) available soil water and VPD affect plant water costs but also plant water demands, as transpiration is the product of *g*_sw_ and VPD; (5) temperature affects Rubisco kinetics, which influences carboxylation costs (as described above), and also the viscosity of water which influences water costs; and finally, (6) elevation affects the saturated vapor pressure of water and hence VPD (influencing water costs), and also gas partial pressures (Körner et al., 1991), ultimately influencing the use of CO_2_ versus O_2_ by Rubisco and therefore carboxylation. Taken together, the optimum balance between resource investments in transpiration and carbon assimilation should thus depend both on soil properties and climate.

Assuming that site properties are the first‐order controls on resource unit costs, typical *V*_cmax 25_–*g*_sw_ and N_area_–*g*_sw_ ratios—and also *C*_i_:*C*_a_—should vary predictably across environmental gradients (and, conversely, there should be convergence in these traits among co‐occurring species). With successive iterations of least‐cost theory, the predictions have shifted from qualitative to quantitative (at least in regards to climate), with support accumulating at regional and global scales. Wright et al. (2003) and Prentice et al. (2014) observed, as predicted, that species from more arid or cooler sites in eastern Australia typically operate with higher N_area_ and *V*_cmax 25_ at a given *g*_sw_, and at lower *C*_i_:*C*_a_. Wang et al. (2017) generated quantitative predictions for the independent effects of site temperature, aridity (VPD), and elevation on *C*_i_:*C*_a_ which were confirmed using a global dataset derived from leaf δ^13^C values (Cornwell et al., 2018). Dong et al. (2017) and Smith et al. (2019) have successfully used least‐cost theory combined with “photosynthetic coordination” theory (Chen et al., 1993; Maire et al., 2012; Von Caemmerer & Farquhar, 1981) to predict climate‐driven patterns in *V*_cmax 25_.

Here, we further investigate the effects of soil properties, primarily pH and total phosphorus (hereafter, P) concentration but also additional proxies for fertility, in driving photosynthetic coordination at a continental scale. In a global study (Paillassa et al., 2020), we reported that plants on neutral to moderately alkaline soils (pH up to 8) had higher *V*_cmax 25_–*g*_sw_, higher N_area_–*g*_sw_, and lower *C*_i_:*C*_a_ than plants on relatively acidic soils (pH as low as 4), and that plants on deeper soils and soils with greater silt content had lower *V*_cmax 25_–*g*_sw_, lower N_area_–*g*_sw_, and higher *C*_i_:*C*_a_ than plants on shallow soils with little silt. These results were interpreted as most likely reflecting lower unit costs for acquiring water on silt‐rich and deep soils, and lower unit costs for acquiring N on higher pH soils. Few studies have investigated the role of soil pH in driving trait coordination, despite its importance for regulating nutrient availability. In that previous study, climate and soil data were derived from global gridded datasets. In the present study, we instead use a combination of measured and gridded soil data and, importantly, we purposefully shift the focus to soil P, a key limiting nutrient for photosynthesis (Domingues et al., 2010; Peng et al., 2021; Reich et al., 2009).

Phosphorus plays a key role in leaf function in relation to P‐rich bioenergetic molecules (ATP, NADP, etc), Calvin–Benson cycle intermediates (e.g., ribulose‐1,5‐bisphosphate), membrane lipids, and nucleic acids. On deeply weathered and infertile soils, including those in Australia but also the tropics, P is a key limiting nutrient for plant productivity, and geographic variation in soil P delineates native vegetation communities (Beadle, 1954, 1966; Kooyman et al., 2017; Laliberté et al., 2014; Vitousek, 1984). Here we address the aforementioned knowledge gaps, combining published and unpublished datasets with de novo photosynthetic measurements, building a comprehensive photosynthetic trait database for Australian native plants (536 species from 67 sites, Figure S1).

**FIGURE 1:**
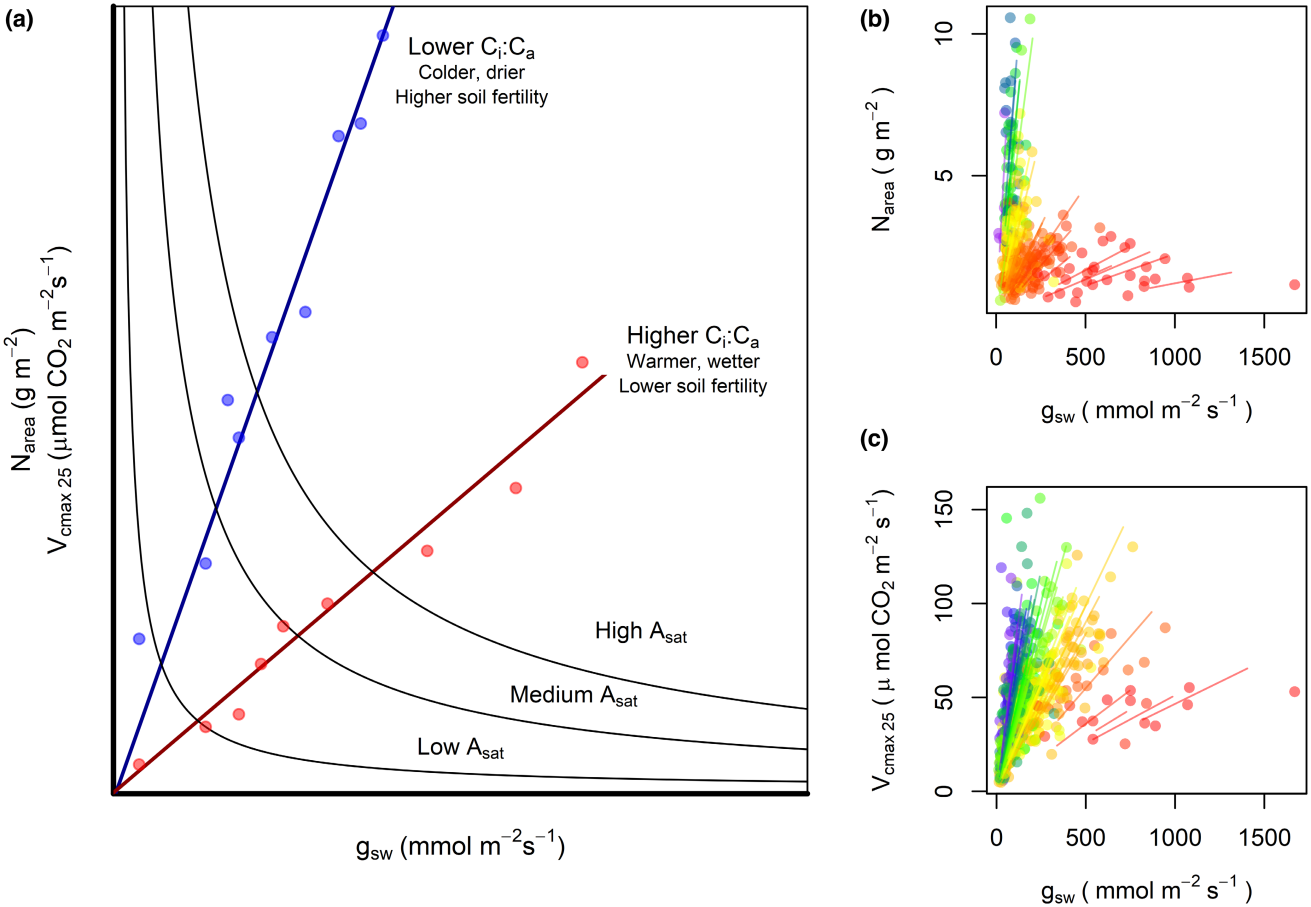
An approach based on least‐cost theory for understanding the co‐optimization of photosynthetic traits in relation to site properties. (a) The theory predicts that the optimal ratio of water and nitrogen (N) use during light‐saturated photosynthesis (*A*_sat_) depends on their relative costs of acquisition and use. Nitrogen use is represented by leaf N content per unit area (N_area_) and carboxylation capacity (*V*_cmax 25_). Water use is represented by stomatal conductance (*g*_sw_). Blue dots represent site conditions where water costs are relatively greater than N costs, or alternatively, where N costs are relatively lower than water costs. *C*_i_:*C*_a_ is the ratio of leaf internal to ambient CO_2_ concentration and mediates the relationship between N use (and carboxylation) and water use. In this study, we found significant site‐level variation in (b) photosynthetic N use versus water use, and (c) carboxylation versus water use, which we quantified using a slope fitted to a set of co‐occurring species at each site, where each point represents a species‐site mean. Each line was “forced” through the origin. Blue and purple tones represent sites with higher water costs and simultaneously lower N costs, while orange and red tones represent lower water costs and simultaneously higher N costs.

Our aims were to understand the manner in which soils—and to a lesser extent climate—have driven the coordination of photosynthetic traits, and to characterize trait–environment relationships, focusing on soil pH, soil total P concentration, mean annual precipitation (MAP), and MAT for the Australian flora. Better regional and global understanding of photosynthetic trait–environment relationships has the potential to improve existing global vegetation models by expanding on the environmental dependencies of traits. We focused on the effects of soil fertility via soil total P concentration and soil pH, both of which presumably influence the unit costs of N and carboxylation more so than water costs, and we tested a number of key predictions (Figure 1a). First, assuming—all else equal—that the unit costs of soil nutrients are lower on higher P soils or higher pH soils, we predicted that plants would increase their investment in N_area_ or *V*_cmax_ relative to *g*_sw_ in these situations, and operate at lower *C*_i_:*C*_a_. We note that while extremely high pH soils reduce soil nutrient availability (Lambers & Oliveira, 2019), Australian soils are predominantly acidic compared with other arid regions of the world (Slessarev et al., 2016). Second, we predicted the same trait shifts (higher *V*_cmax_–*g*_sw_, higher N_area_–*g*_sw_, and lower *C*_i_:*C*_a_) in arid compared with wetter sites and on relatively colder compared with warmer sites. These predictions arise from the assumption that the unit cost of water is greater at low rainfall and high VPD, and that temperature affects the unit costs of both carboxylation and photosynthetic water use (Prentice et al., 2014), as described above. The results from this study will be of global significance, as they will clarify whether trait coordination patterns observed at a global scale are consistent at a continental scale, in the context of locally relevant soil properties.

## 2 MATERIALS AND METHODS

### 2.1 Study system

Australia is highly suited for this line of inquiry as there is wide environmental variation in both soils and climate. The central portion of the continent (ca. 70% by land area) is arid to semi‐arid, while coastal regions vary from Mediterranean in the south to southwest, cool temperate in the south, temperate to tropical in the east, and wet–dry tropics in the far north. Although Australian soils are on the whole ancient and nutrient‐poor (He et al., 2021; Kooyman et al., 2017; Viscarra Rossel & Bui, 2016), higher‐nutrient soils punctuate the landscape (de Caritat et al., 2011; Viscarra Rossel & Bui, 2016) and the Great Dividing Range, which runs 3500 km north to south, approximately parallel to the east coast of Australia, divides the mesic coastal regions from the arid interior. Furthermore, although much of Australia has acidic soil, calcareous soils with high pH are also present across wide areas (de Caritat et al., 2011), for example in southern Australia, resulting from repeated marine incursions beginning in the Miocene era (Northcote & Wright, 1982; Taylor, 1994). In this study, the majority of the sites were on acidic soils with low soil nutrient availability (Table S1), which is representative of Australia but also relevant to other, similarly leached regions of the world.

### 2.2 Field data collection

Leaf trait data were collected on woody and non‐woody plant species at three sites between December 2018 and March 2019: Kidman Springs Research Station (tropical savanna, sampled during the wet season; Northern Territory), Royal National Park (subtropical rainforest; New South Wales), and Mount Keira (subtropical rainforest; New South Wales). Latitude, longitude, and climate data for these sites can be found in Table S1. These sites were chosen to increase the number of samples within sites of moderately high total soil P concentrations and moderately high soil pH (Figure S2), compared with site coverage in our compilation of literature data, described below. Ten soil samples (to 30 cm depth) were collected at each site and air‐dried prior to laboratory analyses (CSPB laboratory in Bibra Lake WA, Australia) of soil pH in CaCl_2_ solution (Rayment & Lyons, 2011) and total soil P concentration via colorimetry, following Kjeldahl digestion (Rayment and Lyons Method 9A3b).

We sampled seven to 28 species per site, randomly selecting three to eight individuals per species and focusing on dominant woody and non‐woody species (excluding C_4_ plants). Photosynthetic traits were measured using a Li‐6800 gas exchange system (Li‐Cor Biosciences). Survey‐style gas exchange measurements were made between 0800 and 1400 h on one leaf per plant. Young but fully expanded, undamaged leaves were sampled from the most sun‐exposed portion of each canopy. We measured light‐saturated (photosynthetic photon flux density of 1800 μmol m^−2^ s^−1^) photosynthesis per unit area (*A*_area_, μmol CO_2_ m^−2^ s^−1^) at an atmospheric CO_2_ concentration of 400 μmol mol^−1^, allowing leaves to remain in the chamber for several minutes. Leaf temperatures were initially set to 25°C, although in many cases the temperature had to be increased above this to prevent condensation in the cuvette. Mean leaf temperature was 29°C with 95% of measurements made between 25 and 35°C; relative humidity varied between 40 and 80%. We also recorded stomatal conductance to water vapor (*g*_sw_, mmol m^−2^ s^−1^) associated with light‐saturated photosynthesis, and the ratio of internal to ambient CO_2_ concentration (*C*_i_:*C*_a,_ unitless). We note that gas exchange rates are sensitive to plant water status and can exhibit pronounced temporal (e.g., diurnal, seasonal) variation. By measuring photosynthesis and stomatal conductance in light‐saturated leaves at a controlled temperature and humidity, we reduced the amount of variation in the data by creating favorable conditions for photosynthesis inside the cuvette.

We also collected five or more outer canopy leaves per plant, sampling from multiple branches up to 10 m above the ground, using an extendable pole pruner. Leaves were scanned to estimate leaf area, dried at 60°C for a minimum of 72 h and weighed to calculate leaf mass per area (LMA, g m^−2^). Samples were analyzed for leaf N concentration (% mass basis) by the Stable Isotope Core Laboratory at Washington State University, USA using an elemental analyzer (ECS 4010, Costech Analytical). Leaf N per area (N_area_; g m^−2^) was calculated as N_area_ = N_mass_ × SLA^−1^.

### 2.3 Data compilation

#### 2.3.1 Trait data

We compiled field‐measured photosynthetic trait data from published and unpublished studies that employed similar standard methods to those described above, that is, light‐saturated photosynthesis measured on young but fully expanded, undamaged “sun” leaves at ambient atmospheric CO_2_ concentration, and relative humidity between 40 and 80%. See Table S2 for a full list of source publications, noting that some of the trait data included herein are not published. Further details regarding our field methods can be found below. For inclusion, a dataset had to contain field‐measured *A*_sat_, *g*_sw_, and *C*_i_; where available we also extracted data for leaf temperature (T_leaf_), LMA, and N_area_. We estimated carboxylation capacity at a standardized temperature of 25°C (*V*_cmax 25_) following the one‐point method (De Kauwe et al., 2016), which utilizes T_leaf_, *A*_sat_, and *C*_i_. We consulted the original publication or contacted the data owners to determine the appropriate leaf temperature for studies where T_leaf_ was not reported. If *V*_cmax 25_ from a CO_2_‐response (A‐*C*_i_) curve was provided, we used these data rather than estimating *V*_cmax 25_ via the “one‐point method” (De Kauwe et al., 2016); 179 measurements, or 6% of the original dataset. To ensure consistency in approach to estimating *V*_cmax_, R_day_ (CO_2_ evolution from mitochondria in the light) was estimated as 1.5% of *V*_cmax_, following De Kauwe et al. (2016), rather than from reported estimates of field‐measured leaf “dark” respiration (*R*_*d*_, which were relatively scarce among the compiled datasets).

We visually inspected the data to find obvious errors (e.g., trait values <0; *C*_i_:*C*_a_ >1) and outliers, conservatively excluding from the analyses any observations with *V*_cmax 25_ > 500 μmol CO_2_ m^−2^ s^−1^, and *g*_sw_ > 3000 mmol m^−2^ s^−1^. This resulted in the exclusion of nine observations (seven for *V*_cmax 25_, two for *g*_sw_). These cut‐offs were based on previously published studies (Smith et al., 2019; Wright, Reich, et al., 2004).

In combination with the de novo field measurements described above, we amassed a trait dataset for 3765 individuals of 528 species (85 families), sampled from 67 study sites (Figure S1 and Table S1). One hundred and fifty‐two species occurred at more than one site. On average, 11 species were sampled per site, although this varied widely (Table S1). Species‐mean trait values were calculated at each site, although subspecies were kept separate, when reported. Taxonomy followed The Plant List (accessed via http://www.plantminer.com/). Thirteen individuals could not be identified beyond the genus level but were still included, and 20 species had names that are taxonomically unresolved in The Plant List. The final dataset included a variety of growth forms (mostly trees and shrubs but 28 herbaceous species, or 4.5% of the dataset), primarily evergreen species, no winter‐deciduous species, some drought‐deciduous species such as *Toona ciliata* and *Melia azedarach*, and a mixture of N_2_‐fixing species (mostly Fabaceae but also Casuarinaceae and Zamiaceae) and non‐N_2_‐fixing species (84% of the dataset). There were 10 gymnosperm species, distributed among five families (Araucariaceae, Cupressaceae, Zamiaceae, Podocarpaceae, and Pinaceae). The original data compilation included five C_4_ species from the genus *Atriplex* and *Triodia* (Amaranthaceae and Poaceae, respectively) but these were excluded from calculations of *V*_cmax_ as the one‐point method is based on the Farquhar et al. (1980) model of C_3_ photosynthesis.

#### 2.3.2 Climate and soil data

Long‐term averages (1982–2002) of climate data (Table S3) were obtained for each site from the ANUClimate model (Hutchinson et al., 2009) and TERN Ecosystem Modelling and Scaling Infrastructure (eMAST) data products (Hutchinson et al., 2009; Xu et al., 2015), both of which provide Australia‐wide coverage at 0.01° spatial resolution, 1970–2012 (https://www.tern.org.au/). We include a total of 21 soil and climate properties. Across the 68 study sites, MAT varied from 9.25 to 27.6°C, and MAP from 260 to 4390 mm (Figure S2).

We had field‐measured data for soil total P concentrations from 34 sites and for soil pH (CaCl_2_) from 28 sites. Otherwise we extracted modelled estimates of soil total P concentration and pH (CaCl_2_) from the TERN Soil and Landscape Grid of Australia (Grundy et al., 2015; Viscarra Rossel et al., 2014) (https://data.csiro.au/), which offers Australia‐wide gridded data at a resolution of 3 arc s (ca. 90 × 90 m pixels). We also extracted additional soil properties known to influence soil fertility, including soil texture, soil organic matter concentration, and cation exchange capacity (Table S3). In the combined dataset, soil total P concentration varied from 28.8 to 3790 ppm (mg kg^−1^), and pH from 3 to 9. There were two sites with exceptionally high (measured) soil P concentrations (Dorrigo National Park, NSW and Curtain Fig National Park, QLD); without these sites, maximum soil P concentration was 1786 ppm.

### 2.4 Statistical analyses

We report results from all models with *p* < .1, noting those with .05 < *p* < .10 as “marginally significant.” All statistical analyses were carried out in R version 3.5.3 (R Development Core Team, 2017).

#### 2.4.1 Testing predictions from least‐cost theory

*V*_cmax 25_–*g*_sw_ and N_area_–*g*_sw_ relationships at each site were summarized as standardized major axis (SMA) slopes fitted with no intercept term (i.e., “forced” through the origin), using untransformed data. These slopes, therefore, represent the average ratios of *V*_cmax 25_–*g*_sw_ and N_area_–*g*_sw_ at each site (Wright et al., 2003). We conducted a slope heterogeneity test to assess site differences, using “SMATR” for R (Warton et al., 2006). Sites with low replication (<3 species per site) were left out from this analysis (two sites for *V*_cmax 25_ and three sites for N_area_), resulting in 58 *V*_cmax 25_–*g*_sw_ slopes and 39 N_area_–*g*_sw_ slopes.

Next, bivariate and multiple linear regression analyses were used to quantify the influence of soil and climate properties on *C*_i_:*C*_a_ as well as the *V*_cmax 25_–*g*_sw_ and N_area_–*g*_sw_ slopes. We tested how these traits varied in response to a total of 21 abiotic variables using bivariate regressions but were unable to include the full suite of predictors in the multiple regression due to multicollinearity and a lack of statistical power. For example, while VPD is often considered an important variable driving photosynthetic trait coordination (Paillassa et al., 2020), in this study it was highly correlated with both MAP and MAT. Similarly, soil pH was correlated with soil N concentration, and soil P and N concentrations were correlated with one another (Figure S3). Therefore, we reduced the set of predictors in the multiple regression to focus on soil pH and soil P concentration, which were not correlated (Figure S3), and also MAP and MAT, which were only weakly (positively) correlated (Figure S2). We selected soil P rather than soil N because soil P is a more strongly limiting soil nutrient for plants within Australia (Beadle, 1954, 1966). Soil pH and soil P were independently correlated with MAT and MAP in opposing directions: soil P concentration was negatively (albeit weakly) correlated with MAT and positively correlated with MAP, whereas soil pH was positively correlated with MAT and negatively correlated with MAP. In other words, relatively colder sites and sites with higher mean annual precipitation had a lower soil pH and higher soil total P concentration, on average (Figure S2). With the exception of *C*_i_:*C*_a_ in the multiple regression, the dependent and independent variables were log_10_‐transformed prior to analyses to meet assumptions of normality.

From multiple regression analyses, we report the beta values for each predictor, that is, the regression weights for standardized variables, representing the change in the response variable (in standard deviations) associated with a change of one standard deviation in a given predictor, other predictors being held constant (Courville & Thompson, 2001; Pedhazur, 1997). These partial effects were visualized with “added variable” (partial regression) plots, created using the avPlots function in the “car” package. Beta weight values (hereafter, β) were calculated using the regr function in the “yhat” package.

We also ran the above analyses after excluding species that were presumed to fix N_2_ (Fabaceae, Casuarinaceae, Zamiaceae) as, on average, these species had notably higher leaf N_mass_ and N_area_ than non‐fixing species (*p* < .001, Figure S4). However, the results (Figure S5) changed little compared with those from main analyses, the key difference being that soil P exerted a stronger, positive effect on the N_area_–*g*_sw_ relationship. Our overall conclusions were not affected therefore these results are not discussed further.

#### 2.4.2 Quantifying climate and soil effects on photosynthetic traits

Climate and soil effects on individual photosynthetic traits were quantified via ordinary least squares (OLS) linear regressions, implemented using the lm function in base R. We investigated relationships between the four focal plant traits (*C*_i_:*C*_a_, *g*_sw_, *V*_cmax 25_, and N_area_) and all 21 soil and climate variables. For this analysis, and for the subsequent partial regression analysis, we included additional traits known to covary with N_area_, including P_area_, LMA, and *A*_area_. We also included photosynthetic phosphorus and nitrogen‐use efficiency, PPUE and PNUE, respectively. In preliminary analyses, we tested quadratic fits between the focal traits and soil pH, finding that the quadratic models for *C*_i_:*C*_a_, N_area_, and *V*_cmax 25_ had lower AIC (>2) than the linear models but added very little explanatory power: *R* ^2^ values of quadratic models ranged from 0.04 to 0.17, with a relative increase in *R* ^2^ ≤ 0.02 for all traits. There was no improvement in the model fit for *g*_sw_. Because our study sites were dominated by acidic soils (pH <7) we had no a priori reason to expect non‐linear relationships between soil pH and nutrient availability, as typically occurs when comparing strongly acidic to strongly alkaline soils (Maire et al., 2015). Therefore, we did not expect non‐linear relationships between soil pH and the focal traits and retained linear fits for all relationships. Leaf traits and abiotic variables were log_10_‐transformed prior to the statistical analyses to satisfy assumptions of normality and homoscedasticity of the residuals.

We also evaluated trait–environment relationships using partial regression analyses on models that included either the four key predictors above (soil P, soil pH, MAP, and MAT) or seven predictors (soil P, soil pH, soil N, MAP, MAT, VPD, and radiation), which were selected because they are known to influence photosynthetic traits. Correlations between abiotic variables were visualized using the corrmat function in the “corrmat” package.

## 3 RESULTS

### 3.1 Trait variation

In the species‐mean dataset, *V*_cmax 25_ varied ca. 27‐fold (from 5.8 to 156 μmol m^−2^ s^−1^, *n* = 636), *g*_sw_ varied ca. 150‐fold (from 11.1 to 1670 mmol m^−2^ s^−1^; *n* = 664), N_area_ ca. 19‐fold (0.55 to 10.6 g m^−2^; *n* = 430), and *C*_i_:*C*_a_ varied ca. fourfold (from 0.22 to 0.96; *n* = 665). The notably wider range in *g*_sw_ was due to one exceptionally high value for *Eucalyptus miniata* from Eamus and Prichard (1998). Excluding this *g*_sw_ would have resulted in a 90‐fold variation; however, we had no basis on which to exclude this value. If variation in traits was compared in terms of the ratio of 97.5th to 2.5th percentiles, rather than maximum/minimum, variation in *g*_sw_ was comparable to that in other traits (ca. 22‐fold). By comparison, the ratio of 97.5th to 2.5th percentiles for *g*_sw_ was ca. 23 in the global photosynthetic trait dataset of Maire et al. (2015).

### 3.2 Bivariate tests of least‐cost theory

**FIGURE 2:**
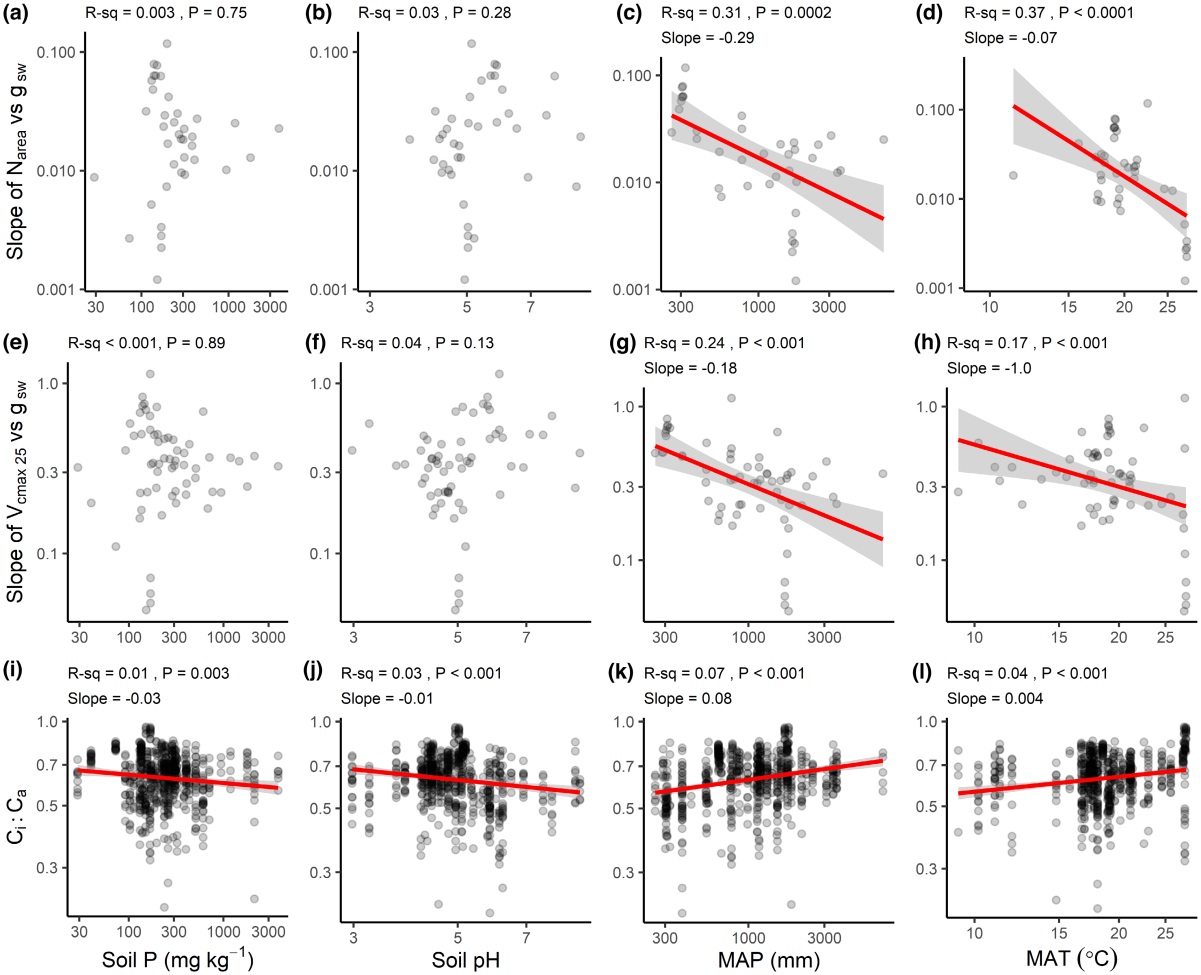
Linear regression plots of soil and climate effects on the (a)–(d) slope relationship between leaf nitrogen concentration (N) on an area basis, N_area_, and stomatal conductance, *g*_sw_, and the (e)–(h) slope relationship between photosynthetic carboxylation, *V*_cmax 25_, and *g*_sw_. (i)–(l) Relationship between *C*_i_:*C*_a_ and environmental variables. (a), (e), (i) Soil total phosphorus (Soil P, mg kg^−1^) concentration, (b), (f), (j) Soil pH, (c), (g), (k) Mean annual precipitation (MAP, mm), and (d), (h), (l) Mean annual temperature (MAT, °C). Red lines represent trend lines with 95% confidence intervals in gray and are only shown for statistically significant (*p*‐values <.05) relationships. Notice the logarithmic scale to the axes. See Figure 3 for partial regressions.

N_area_–*g*_sw_ and *V*_cmax 25_–*g*_sw_ slopes varied widely across sites (slope heterogeneity *p* < .001, Figure 1b,c) where steeper slopes indicate that species are operating with higher *V*_cmax 25_ or N_area_ at a given rate of stomatal conductance to water vapor (Figure 1a). Contrary to our expectation, variations in these slopes were not associated with soil P concentration or soil pH in the bivariate regressions (Figure 2), and typically, the soil variables explained less than 5% of the variation in the slopes. Similarly, we found no association between the slope relationships and soil nitrogen (Soil N), bulk density of whole earth (BDW), soil organic carbon (SOC), and the soil textural properties. The only variable that significantly influenced the slope relationships was ECE, which exerted a positive effect on the slopes (Table S4), suggesting that higher ECE increased nutrient availability and reduced nutrient costs relative to water costs.

*C*_i_:*C*_a_ varied with both soil pH and soil P concentration in the expected manner, being lower on average at sites with high soil P concentrations (Figure 2i) or high pH (Figure 2j). *C*_i_:*C*_a_ also decreased with increasing ECE, increasing SOC, and increasing clay content (and increased with increasing silt and sand content) (Table S4), supporting our predictions (Figure 1a).

With regards to climate, the N_area_–*g*_sw_ and *V*_cmax 25_–*g*_sw_ slopes were generally steeper at drier sites (Figure 2c,g) and at colder sites (Figure 2d,h), as predicted. For example, N_area_–*g*_sw_ slopes were ca. sixfold steeper at 300 mm MAP than at 3000 mm MAP (0.04 vs. 0.007, respectively), and *V*_cmax 25_‐*g*_sw_ slopes were ca. threefold steeper (0.52 vs. 0.19, respectively). From the bivariate regressions, MAP explained 31% and 24% of the variation in N_area_–*g*_sw_ and *V*_cmax 25_–*g*_sw_ slopes, respectively (Table S4, Figure 2c,d). MAT explained 37% and 17% of the variation in N_area_–*g*_sw_ and *V*_cmax 25_–*g*_sw_ slopes, respectively (Figure 2d,h).

Also as predicted, species at drier sites and at relatively colder sites operated at lower *C*_i_:*C*_a_ (Figure 2k,l). Using a standard moisture index, the ratio of MAP to potential evapotranspiration (Thornthwaite, 1948), gave similar results to using MAP alone (Table S4).

In general, climate variables explained a significantly greater percentage of the variation in the N_area_–*g*_sw_ and *V*_cmax 25_–*g*_sw_ slopes (0.005 ≤ *R* ^2^ ≤ 0.64) than did the soil variables (0.004 ≤ *R* ^2^ ≤ 0.24). Similarly, a greater amount of variation in *C*_i_:*C*_a_ was explained by climate (max *R* ^2^ = 0.12) than by soil (max *R* ^2^ = 0.08).

### 3.3 Multiple regression tests of least‐cost theory

**FIGURE 3:**
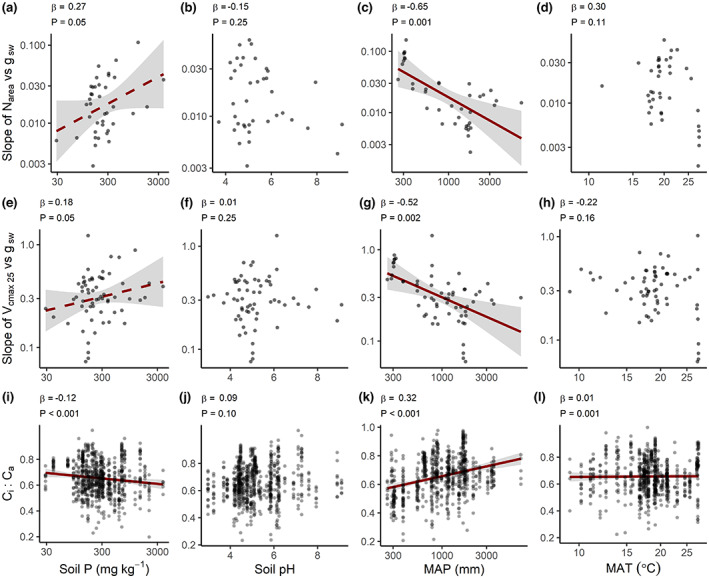
Partial regression plots from multiple linear regression of soil and climate effects on the (a)–(d) slope relationship between leaf nitrogen concentration (N) on an area basis, N_area_, and stomatal conductance, *g*_sw_, and the (e)–(h) slope relationship between photosynthetic carboxylation, *V*_cmax 25_, and *g*_sw_. (i)–(l) Relationship between *C*_i_:*C*_a_ and environmental variables. (a), (e), (i) soil total phosphorus (soil P, mg kg^−1^) concentration, (b), (f), (j) soil pH, (c), (g), (k) mean annual precipitation (MAP, mm), and (d), (h), (l) mean annual temperature (MAT, °C). Points in gray represent partial regressions with standard errors in gray and dark red lines are shown only for statistically significant relationships, where solid lines have *p* < .05 and dashed lines are marginally significant (.05 < *p* < .10). *p*‐values above each panel indicate the statistical significance of each variable in the multiple regression. Higher β values indicate a stronger effect size, where β values are the regression weights for standardized variables and represent the change in the slope value (in standard deviations) associated with a change of one standard deviation in a predictor while holding constant the value(s) of the other predictor(s).

Multiple regression analyses revealed some distinct patterns from the bivariate regressions (Figure 3). Together, the four environmental variables explained 52% of variation in N_area_–*g*_sw_ slopes, 36% of variation in *V*_cmax 25_–*g*_sw_ slopes, and 14% of variation in *C*_i_:*C*_a_. Comparing standardized regression coefficients (β values in Figure 3), MAP affected photosynthetic trait coordination more strongly than the three other environmental variables. The effect sizes for soil P concentration were of similar or slightly stronger magnitude to those for MAT, and notably weaker than the precipitation effects.

After controlling for variation in other predictors via partial regression, the effect of soil P concentration on the N_area_–*g*_sw_ and *V*_cmax 25_–*g*_sw_ relationship slopes became stronger than what we observed in the OLS regression (i.e., 0.05 < *p* < .10; Figure 3a,e). The soil P concentration effect on *C*_i_:*C*_a_ (Figure 3i) was again negative, even when controlling for variation in MAT, MAP, and soil pH. These effects of soil P concentration were all in the predicted direction (Figure 1a). After controlling for variation in other predictors, soil pH still showed no association with N_area_–*g*_sw_ and *V*_cmax 25_–*g*_sw_ relationship slopes (Figure 3b,f) or *C*_i_:*C*_a_ (Figure 3j).

For both sets of slopes, models including all four predictors indicated that the MAP effect was strongly negative (in terms of β), and was stronger than that of MAT, soil P concentration and soil pH (Figure 3). That is, at a given MAT, soil P concentration or soil pH, species at drier sites typically operated with higher *V*_cmax 25_ or N_area_ at a given *g*_sw_ (Figure 3c,g), and also typically had lower *C*_i_:*C*_a_ (Figure 3k)—all trends consistent with the expectation that savings on photosynthetic water use can be achieved via increased investment in the N‐rich carboxylating enzyme, Rubisco. The MAT effects on the N_area_–*g*_sw_ and *V*_cmax 25_–*g*_sw_ slopes in the bivariate regressions were no longer apparent once other environmental variables were controlled (Figure 3d,h). By contrast, a positive MAT effect on *C*_i_:*C*_a_ was observed when controlling for other variables, as was the case in the bivariate analysis (Figure 3l).

### 3.4 Trait–environment relationships

**FIGURE 4:**
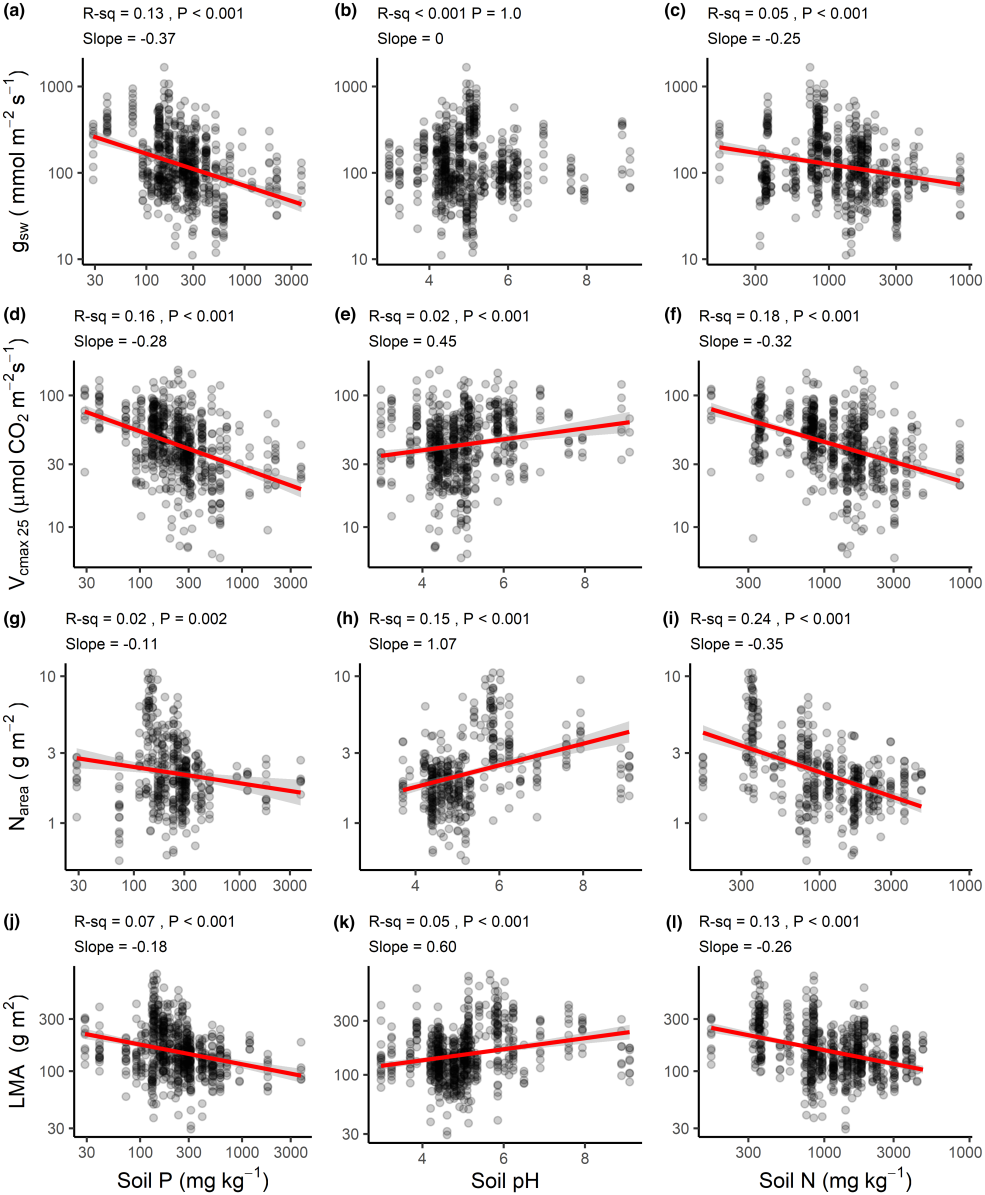
Trait–soil relationships from bivariate linear regression analysis. Points represent species‐site means. All axes (except soil pH) have been log_10_‐scaled. Abbreviations follow those in Table S1 and Figure 3. Red lines represent trend lines with 95% confidence intervals in gray and are only shown for statistically significant (*p*‐values <.05, solid line). In panel (b), the slope coefficient was |<.005|.

We quantified relationships between environmental variables and plant photosynthetic traits including *g*_sw_, *V*_cmax 25_, and N_area_, but also additional traits known to covary with the focal variables (for the full suite of relationships, see Table S4). Species on low‐P and on low‐N soils tended to have higher *g*_sw_, N_area_, and *V*_cmax 25_ (Figure 4), whereas species on high pH soils (which in this dataset are expected to have higher soil nutrient availability) had higher *V*_cmax 25_ and higher N_area_ but exhibited no difference in *g*_sw_ (Figure 4b). The higher *V*_cmax 25_ and N_area_ on low‐P soils were likely driven by higher LMA on low‐P soils (Figure 4j, Table S4), as N_area_ was positively correlated with both LMA _25_ (*r* = 0.75, *p* < .001) and *V*_cmax 25_ (*r* = 0.37, *p* < .001). Soil P concentration explained the highest percentage of the variation in *V*_cmax 25_ (*R* ^2^ = 0.16) whereas soil N concentration explained the highest percentage of the variation in N_area_ (*R* ^2^ = 0.24). On average, for a tenfold decrease in soil P concentration, *V*_cmax 25_ increased 1.5‐fold and *g*_sw_ twofold. N_area_ showed a significant association with soil P concentration but with little explanatory power (*R* ^2^ = 0.02; Figure 4g).

**FIGURE 5:**
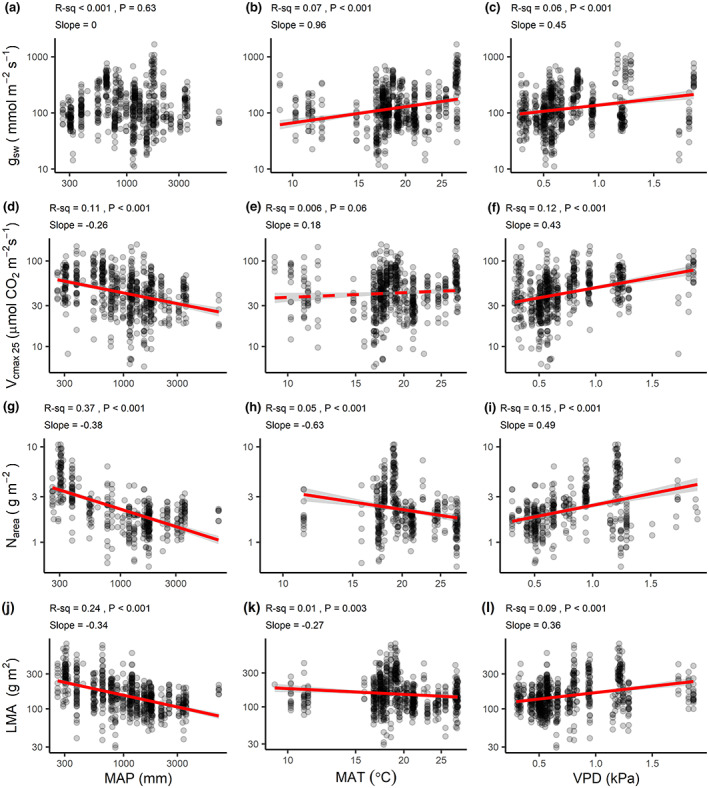
Trait–climate relationships from bivariate linear regression analysis. Points represent species‐site means. All axes (except soil pH) have been log_10_‐scaled. Abbreviations follow those in Table S1 and Figure 3. Red lines represent trend lines with 95% confidence intervals in gray and are only shown for statistically significant (*p* < .05, solid line, 0.05 < *p* < .10, dashed line). In panel (a), the slope coefficient was |<.005|.

Species at low‐MAP sites (especially at MAP <1000 mm) tended to have higher N_area_ (*R* ^2^ = 0.37; Figure 5g), which was by far the strongest correlation in this part of our analysis. The higher N_area_ at low rainfall corresponded (as expected) to higher *V*_cmax 25_ (*R* ^2^ = 0.11; Figure 5d). By contrast, *g*_sw_ showed no relationship with MAP (Figure 5a). The N_area_–MAP scaling slope of −0.39 indicates that for a tenfold decrease in MAP, N_area_ increased nearly 2.5‐fold, on average. On average, there was a 1.5‐fold increase in *V*_cmax 25_ over this same interval in MAP (log–log slope = −0.26). Species at warmer sites typically had higher *g*_sw_ but lower N_area_ (Figure 5b,h), consistent with the predicted and observed MAT effect on N_area_–*g*_sw_ slopes (Figure 1, Figure 3). That said, there was pronounced scatter in these relationships (0.05 ≤ *R* ^2^ ≤ 0.07). *V*_cmax 25_ showed a marginally significant relationship with MAT (Figure 5f) but with <1% explanatory power.

Of the remaining soil variables (Table S4), BDW (0.10 ≤ *R* ^2^ ≤ 0.21), SOC (0.01 ≤ *R* ^2^ ≤ 0.23), and sand content (0.01 ≤ *R* ^2^ ≤ 0.19) explained the most variation in the focal traits. Of the remaining climate variables, the results were idiosyncratic but VPD explained a significant proportion of the trait variation (0.06 ≤ *R* ^2^ ≤ 0.15), as expected.

The partial regression analysis on the trait–environment relationships (Table S5) was largely similar to the OLS regression with a few exceptions. First, the effect of soil P on N_area_ was positive (rather than negative) when we accounted for the other abiotic variables. Second, there was a significant negative effect of soil pH on *g*_sw_ and on *C*_i_:*C*_a_, where previously these relationships were not statistically significant.

## 4 DISCUSSION

Despite the critical role of photosynthesis in driving the carbon cycle of terrestrial ecosystems, we understand relatively little about how soil fertility influences the coordination of photosynthetic traits, and the relative effects of soil versus climate. Here, we report the effects of a globally relevant, limiting soil nutrient, phosphorus (P), on photosynthetic trait coordination. Previously, for four sites in Australia, we reported trait shifts in relation to site temperature and aridity that were consistent with predictions from least‐cost theory (Prentice et al., 2014; Wright et al., 2003). In a global study (Paillassa et al., 2020), we then examined the interactive effects of soil and climate, focusing on pH and soil texture as indices of fertility. Here we expand on the Australian study, including hundreds more species from many more sites (67), a much wider range of climate variables and, very importantly, we extend the analyses to soil nutrients.

### 4.1 Climate effects

Using the largest Australian photosynthetic trait dataset to date, we generally observed that climate effects were stronger than soil effects. We report strong climate‐driven trait shifts in line with previous studies and in line with our predictions. Most notably, with lower MAP we observed higher N_area_ and *V*_cmax 25_ at a given *g*_sw_, higher N_area_ and *V*_cmax 25_ overall, and lower *C*_i_:*C*_a_. Although *g*_sw_ was not influenced by site precipitation, higher N_area_ and *V*_cmax 25_ drove the steeper N_area_–*g*_sw_ and *V*_cmax 25_–*g*_sw_ slope relationships across the precipitation gradient. Steeper *V*_cmax 25_–*g*_sw_ and N_area_–*g*_sw_ relationships at drier sites underlie the lower *C*_i_:*C*_a_ in these places, the higher carboxylation capacity (at a given *g*_sw_) drawing leaf‐internal CO_2_ down to lower concentrations. The lack of patterning of *g*_sw_ with respect to MAP accords with “global” results from Wright, Reich, et al. (2004) and Murray et al. (2019, 2020). The uncoupling of *g*_sw_ from MAP is interesting, as VPD is typically higher on more arid sites and *g*_sw_ increased with VPD (Table S4), indicating higher transpiration rates when stomata are open. Similar to the present study, global (Paillassa et al., 2020; Wang et al., 2017) and regional studies (Bloomfield et al., 2019; Cernusak, Hutley, et al., 2011; Cochrane et al., 2016; Wright et al., 2001) have reported lower *C*_i_:*C*_a_, higher *V*_cmax 25_,and higher leaf nutrient concentrations (especially per unit area) in drier habitats.

Least‐cost theory predicts that MAT affects both water and carboxylation costs. In this study MAT effects matched our predictions but were weaker than those of MAP. Both the MAT and MAP effects were stronger than soil effects—at least in the bivariate relationships (see 4.2). These findings suggest that environmental variables that solely affect the unit costs of N (and carboxylation) exert weaker effects on photosynthetic trait coordination than do environmental variables that influence the unit costs of water (i.e., MAP) or influence the costs of both water and carboxylation (i.e., MAT). Interestingly, we also observed weaker effects of MAT relative to MAP on the *V*_cmax 25_–*g*_sw_ and N_area_–*g*_sw_ relationships—but not *C*_i_:*C*_a_—when considered in a multiple regression framework. The weaker effect size of MAT in the multiple regression likely resulted from collinearity between MAT and either soil pH (*r* = 0.22) or MAP (*r* = 0.25), the latter of which are often confounded, and can have compounding effects on plant functional traits. For example, globally, species growing on relatively dry sites tend to have small leaves when the mean temperature of the warmest month (TWM) is high, whereas species on wetter sites typically have larger leaves when TWM is high (Wright et al., 2017). From bivariate regressions, we also found that seasonality in temperature strongly (positively) influenced the N_area_–*g*_sw_ and *V*_cmax 25_–*g*_sw_ slope relationships (Table S4), perhaps suggesting that more seasonal environments have higher water costs.

### 4.2 Soil effects

Plants have various strategies that enhance their ability to cope with drought and nutrient deficiency, two properties that characterize much of the Australian continent. Indeed, a significant proportion of Australian plants possess scleromorphic long‐lived leaves with low mass‐based nutrient concentrations (Beadle, 1966; Lambers et al., 2010; Lamont, 1982; Specht, 1969; Wright et al., 2002; Wright, Groom, et al., 2004) and highly proficient nutrient resorption (Wright & Westoby, 2003). That is, most Australian plant species are generally positioned toward the “slow” end of the leaf economics spectrum (Wright, Reich, et al., 2004).

In this study we considered the effects of soil fertility via soil total P concentration and soil pH, both of which presumably influence the unit costs of N and carboxylation more so than water costs. Soil P concentration is a long‐term site property that is strongly determined by parent material and is widely used as an indicator of soil P status in Australian ecology (Beadle, 1954, 1966; Fonseca et al., 2000; Kooyman et al., 2017). In contrast to soil total N concentration, which is quite stable across time, soil N and P availability can vary seasonally and also with plant nutrient‐acquisition strategies, often reflecting root morphology, the tendency for carboxylate release and associations with mycorrhizal fungi (Lambers & Oliveira, 2019; Richardson et al., 2005; Turner, 2008). In the bivariate analyses (Figure 2), *V*_cmax 25_ and *g*_sw_ showed clear negative relationships with soil P concentration (*R* ^2^ = 0.13–0.16) and the soil P effect was far greater than the soil pH effect overall. Because all of *g*_sw_, N_area,_ and *V*_cmax 25_ increased as soil P decreased, it makes sense that their ratios (the N_area_–*g*_sw_ and *V*_cmax 25_–*g*_sw_ slopes) show little pattern over soil P gradients. The negative relationship between N_area_ and soil P in the OLS regression resulted from LMA being typically higher on low‐P soils (Table S4) and in this study, there was a positive relationship between LMA and N_area_ (*r* = 0.75, *p* < .001, results not shown) and between N_area_ and *V*_cmax 25_ (*r* = 0.37, *p* < .001, results not shown). We note, however, that the relationship between N_area_ and soil P became positive when we accounted for the effects of soil pH, MAP, and MAT (Table S5), which likely reflects the strong, negative effect of MAP on LMA and therefore N_area_. A positive relationship between N_area_ and soil P was also observed in partial residual plots generated by Peng et al. (2021), which utilized a global dataset that included Australia.

The high *V*_cmax 25_ at low soil P concentration is novel and unexpected, whereas the negative relationship between soil P and *C*_i_:*C*_a_ matched predictions from least‐cost theory (Tables S4 and S5). Least‐cost theory also predicts that all else equal, *C*_i_:*C*_a_ and *V*_cmax 25_ should be inversely related (Wright et al., 2003), which we observed in the present study (*r* = −0.15, *p ≤* .001, results not shown). In contrast to the *V*_cmax 25_–soil P relationship, the *g*_sw_ effect was in line with our expectations: Maire et al. (2015) reported a negative association between plant‐available soil P concentration and *g*_sw_, arguing that nutrient deficiency promotes greater root production, increasing plant‐available water and increasing *g*_sw_ and *C*_i_:*C*_a_. The authors also suggested that stimulation of transpiration (and *g*_sw_) on nutrient‐deficient sites may increase mass flow of soil nutrients to roots, ultimately enhancing leaf N and ultimately, *V*_cmax 25_ [i.e., the mass‐flow hypothesis (Cernusak, Winter, et al., 2011; Cramer et al., 2009; Edwards et al., 1998)]. Because the mobility of P is low compared with that of N, mass flow is more likely to increase N uptake than P uptake and may only increase P supply on P‐impoverished, sandy soils with low P buffering capacity (Cernusak, Winter, et al., 2011; Huang et al., 2017).

Considering the importance of P for leaf metabolism, environmental properties that affect the per‐unit cost of P acquisition from the soil arguably also affect the unit cost of carboxylation, vis‐à‐vis least‐cost theory. The chief way that soil P is more expensive to acquire on low‐P soils is in terms of higher belowground expenditure, for example, greater fine root production, greater expenditure supporting mycorrhizal symbionts, greater expenditure on root exudates that enhance access to recalcitrant pools of soil P (e.g., phosphatases; organic acids released by cluster toots), and greater expenditure on cluster roots (Raven et al., 2018). The latter are especially common in the Australian flora, particularly in the Proteaceae which exhibit very high photosynthetic phosphorus‐use efficiency (PPUE) (Denton et al., 2007; Guilherme Pereira et al., 2019), that is, rapid photosynthetic rates at low leaf P concentrations (Lambers et al., 2012; Yan et al., 2019). In this study, we observed higher PPUE and higher PNUE for plants growing on low‐P soils (Table S4) and higher *V*_cmax 25_ on low‐P soils. High PPUE may be accomplished by shifting allocation away from phospholipids toward galactolipids (the latter being a key component of chloroplast membranes) and sulfolipids that do not contain P (Lambers et al., 2012; Yan et al., 2019) with the transition from young to mature leaves. Interestingly, Australian Proteaceae growing on P‐deficient soils have been shown to have low Rubisco activity but high levels of photosynthesis at low leaf P compared to *Arabidopsis* (Sulpice et al., 2014). The reduction in Rubisco activity likely resulted from a lower abundance of ribosomes and therefore lower rRNA levels, which may constrain the synthesis of proteins, including Rubisco. Thus, Australian plants appear to be well‐adapted to low‐P soils, as they maintain high levels of photosynthesis, high rates of carboxylation, and high photosynthetic nutrient‐use efficiency in these environments.

Soil pH alters the solubility of soil minerals and causes shifts in community composition of soil bacteria (Lauber et al., 2008), which in turn affects nutrient availability. We thus included soil pH alongside soil total P concentration, due to its potential effects on the unit cost of carboxylation. Against expectation, variation in soil pH had negligible effects on the N_area_–*g*_sw_ and *V*_cmax 25_–*g*_sw_ relationships but *C*_i_:*C*_a_ did decrease with increasing pH, albeit weakly (*R* ^2^ = 0.03), as predicted. While the pH effects were weak to negligible, the pattern in *C*_i_:*C*_a_ matched global studies that showed strong modulation of N_area_–*g*_sw_ and *V*_cmax 25_–*g*_sw_ relationships, *C*_i_:*C*_a_ and Δ^13^C via soil pH (Cornwell et al., 2018; Paillassa et al., 2020; Wang et al., 2017). *C*_i_:*C*_a_ is tightly determined by the balance between *V*_cmax 25_ and *g*_sw_, such that the *V*_cmax 25_–*g*_sw_ relationship is a function of the optimal *C*_i_:*C*_a_, which is itself a function of water and N costs (Prentice et al., 2014). Therefore, it is possible that *C*_i_:*C*_a_ better reflects costs associated with soil and climate properties than do the slopes, as it more directly integrates N and water costs. This is evidenced by the observation that *C*_i_:*C*_a_ was sensitive (statistically significant) to all four of the chosen environmental variables whether analyzed in bivariate regressions or multiple regression. Although N_area_ was strongly influenced by soil pH (*R* ^2^ = 0.15), *g*_sw_ was not (Figure 4) and *V*_cmax 25_ was only weakly affected (*R* ^2^ = 0.02). The positive effect of pH on leaf N concentration suggests moderately higher soil nutrient availability in less acidic soils, despite the negligible effect of soil pH on the *V*_cmax 25_–*g*_sw_ and N_area_–*g*_sw_ slopes. Maire et al. (2015) also found no relationship between *g*_sw_ and soil pH in a global study and in general, the soil pH effects in this study were considerably weaker than those reported at global scale (cf. Paillassa et al., 2020).

While the weak pH effects in the present study were unexpected, this suggests that soil pH may not be an especially useful index of nutrient‐acquisition costs in low‐fertility landscapes. We note that the partial regression analyses resulted in a statistically significant negative effect of soil pH on *g*_sw_ and *C*_i_:*C*_a_, the latter of which matched our predictions, indicating strong collinearities among the predictors. Nevertheless, soil pH does not underlie variation in photosynthetic traits within this system to the extent that soil P does. The finding that climate has a greater role in photosynthetic trait coordination than soil pH contrasts with the findings of our companion study (Paillassa et al., 2020), and may reflect the predominance of low pH/low‐nutrient sites in our Australian dataset (only 4 of 67 sites with pH >7).

### 4.3 Implications for global studies

By improving our understanding of photosynthetic trait–environment relationships at the regional and global scale, there is the potential to enhance the conceptual basis and parameterization of global vegetation models. For example, dynamic global vegetation models (DGVMs) rarely incorporate variation in ecophysiological traits within plant functional types (e.g., across species or populations) or include environmental dependencies of traits (Grimm et al., 2017; Scheiter et al., 2013; Verheijen et al., 2013; Yang et al., 2015). We have now demonstrated that soil properties, namely soil P concentration and soil pH (Maire et al., 2015; Paillassa et al., 2020), influence the coordination of ecophysiological traits at a continental scale. These findings support increasing calls for soil properties to be included in vegetation models (Norby et al., 2017) and could be further developed using least‐cost theory.

Within the least‐cost theory framework, the first‐order costs are set by site properties, whole‐plant respiration rates, and tissue chemistry (e.g., leaf N or Rubisco concentration). In the formulation of least‐cost theory by Prentice et al. (2014), optimal *C*_i_:*C*_a_ is proportional to the ratio of two dimensionless parameters, *a* and *b*, which reflect the maintenance respiration costs of transpiration and carboxylation, respectively. Paillassa et al. (2020) re‐expressed the cost functions to incorporate the effects of soil N and water supply, surmising that maintenance respiration costs at a given transpiration rate or carboxylation rate should increase when soil water or nutrients are scarce. But, of course, these are necessary simplifications that do not account for all relevant costs. Most importantly, water and nutrient unit costs are presumably also affected by species life history traits. For example, information regarding inter‐ and intraspecific variation in the ability to acquire soil water or nutrients via alternative allocation or acquisition strategies, including root activity and depth, nutrient‐acquisition strategies (e.g., cluster roots vs. N_2_ fixation vs. mycorrhizal symbioses; Lambers et al., 2008), and wood permeability (Wright et al., 2003), would likely help in the interpretation of within‐site variation in photosynthetic traits. For example, mycorrhizal species may have lower N costs than non‐mycorrhizal species, which would result in higher N_area_ for a given *g*_sw_ in the mycorrhizal species. Regions dominated by mycorrhizal species are thus expected to have species with higher N_area_–*g*_sw_ slopes than regions where such species are absent, even when these occur at similar soil nutrient levels.

Additional considerations are needed before we can quantitatively integrate the effects of concentrations of soil P (or other metrics of fertility) in DGVMs. For example, one can make assumptions about the extent to which different nutrients are *substitutable* [e.g., whether species can “spend” more N belowground by investing in phosphatase enzymes to obtain more soil P (Olander & Vitousek, 2000; Schleuss et al., 2020; Treseder & Vitousek, 2001)], or simply *coordinated*, and specify nutrient exchange rates in a currency that can also be applied to water costs. It is also worth considering how additional soil properties influence soil nutrient costs, as soil texture, which influences both the availability of nutrients and water, seems also important for understanding geographic variation in photosynthetic trait coordination (Paillassa et al., 2020). For example, soils with higher silt content can hold more water than sandy soils, reducing water costs, such that plants typically have higher *g*_sw_ coupled with higher *V*_cmax 25_ on silty soils (Paillassa et al., 2020). In this study, we found that plants growing on silt‐rich soils had higher *g*_sw_ and higher N_area_ and *V*_cmax 25_, but similar slope relationships (Table S4), indicating a proportionate increase in these traits, which canceled out. We also found a positive relationship between soil effective cation exchange capacity (ECE) and the slope relationships, indicating that nutrient costs were lower with increasing ECE, which is positively associated with soil nutrient availability. Lastly, we acknowledge the important role of soil N in other regions of the world, which significantly influenced individual photosynthetic traits (with the exception of *C*_i_:*C*_a_) but not trait coordination in this study system. The negative relationship between leaf N and soil total N, which was also reported by Maire et al. (2015), may result from low plant‐available N if the soil organic matter has a high C:N ratio (Parton et al., 1988). Further consideration of long‐ versus short‐term indices of soil resources is also warranted. Here we focused on evolutionary adaptations to soil nutrient pools rather than on acclimation to soil nutrient availabilities that can vary tremendously over relatively short timescales. Future studies could consider the relative strengths of short‐ and long‐term controls on photosynthetic trait coordination, as this would be useful for quantifying within‐species variation in trait relationships.

## 5 CONCLUSIONS

Rainfall and temperature are expected to change considerably over the coming decades, regionally and globally, altering the availabilities of soil nutrients. While much is known regarding how climate drives variation in photosynthesis, few studies have investigated soil effects, although this is changing. Among our findings, the coordination of photosynthetic traits in response to soil P concentration is especially novel, as it suggests a unique contribution of a limiting soil nutrient that is independent of climate and soil pH. The simple theoretical framework known as least‐cost theory can thus be applied to low‐nutrient regions globally, for example, highly weathered soils and tropical regions, where P limits productivity. By considering the dependencies of plant traits on both climate and soils, we will better understand the proximate and long‐term controls of photosynthesis.

## AUTHOR CONTRIBUTIONS

Ian J. Wright and Andrea C. Westerband planned and designed the study. Andrea C. Westerband carried out the fieldwork in 2018 and 2019 and analyzed the data. Andrea C. Westerband wrote the first draft with significant input from Ian J. Wright. All authors read and contributed to subsequent versions.

## CONFLICT OF INTEREST

The authors declare no conflicts of interest.

## Supporting information


Data S1

## Data Availability

The data that support the findings of this study are openly available in Dryad at https://doi.org/10.5061/dryad.j9kd51cgr.
