# Supplementary material for: Coordination of photosynthetic traits across soil and climate gradients
Source: Glob Chang Biol. 2022 Nov 16;29(3):856–73. doi: 10.1111/gcb.16501 (PMC10098586; doi:10.1111/gcb.16501)
Supplement: Supplementary file 1 — Data S1 [file GCB-29-856-s001.docx]

## Supplementary Information

**Fig. S1** Study site information. Distribution of study sites across the Australian continent (N = 67). Numbers denote approximate number of sites per cluster, in cases where sites are overlapping. See Table S1 for geographic coordinates and dominant soil order at each site.


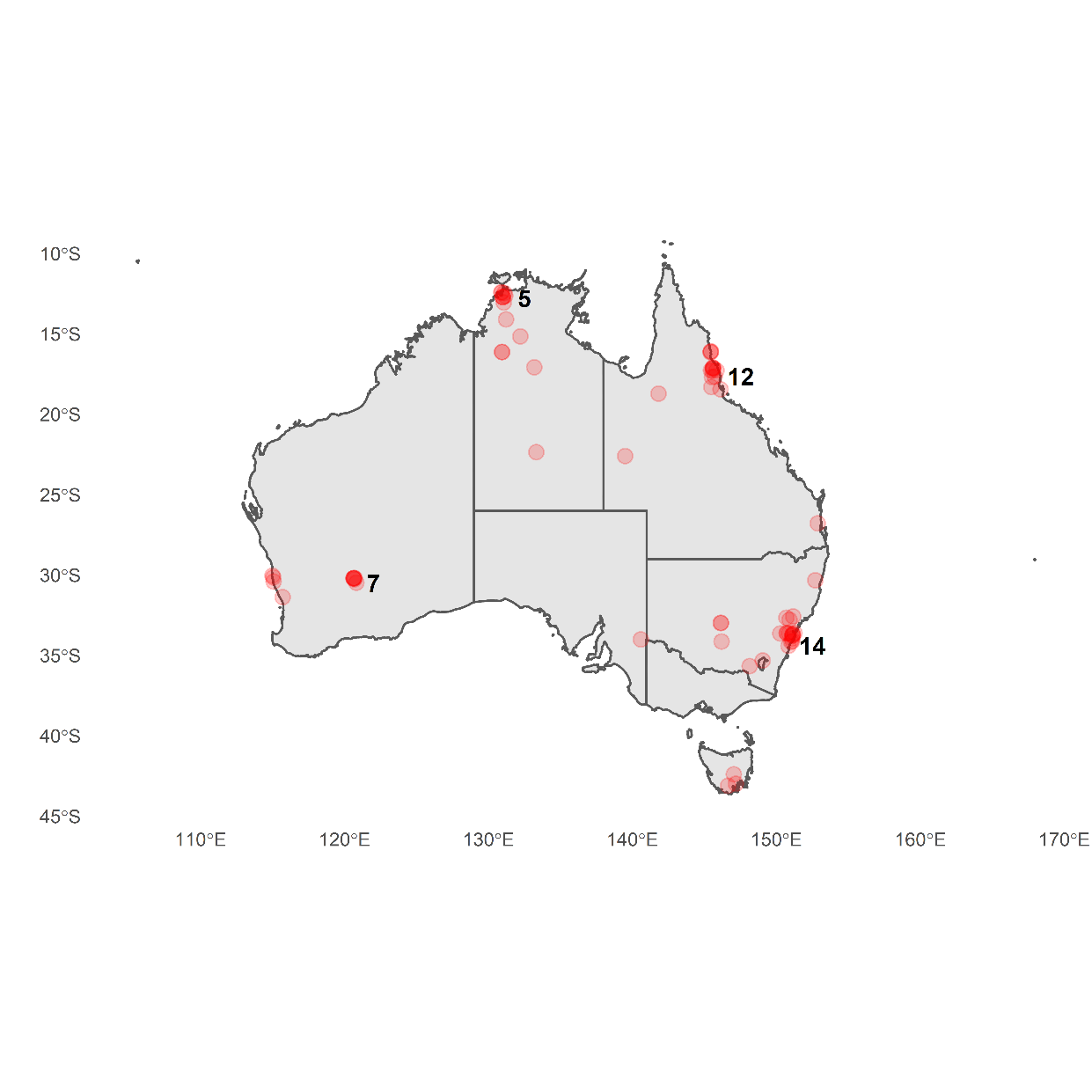


**Table S1** Site properties. Abiotic properties of 67 study sites within Australia (Fig. S1). Site names follow those used in data sources/source publications. Geographical coordinates and dominant soil order are also shown and follow the classification system of the Australian sSoil Resource Information System.

| **Site** | **Soil Order** | **Longitude** | **Latitude** | **MAP (mm)** | **MAT (°C)** | **Soil P (mg kg^-1^)** | **Soil pH** | **Num Species** |
| --- | --- | --- | --- | --- | --- | --- | --- | --- |
| Adelaide River | Rudosols | 131.07 | -13.04 | 1571.3 | 27.3 | 173.6 | 5.1 | 2 |
| Agnes Banks | Rudosols | 150.72 | -33.6 | 774.2 | 17.8 | 381.4 | 4.8 | 3 |
| Alice Mulga | Rudosols | 133.33 | -22.35 | 321.7 | 22.6 | 196.9 | 5.1 | 15 |
| Atherton | Rudosols | 145.46 | -17.26 | 1305.6 | 20.1 | 709.5 | 5.4 | 9 |
| Bago | Rudosols | 148.15 | -35.66 | 1510.6 | 9.3 | 504.9 | 4.3 | 4 |
| Berrimah | Rudosols | 130.92 | -12.42 | 1787.6 | 27.4 | 152.2 | 4.9 | 5 |
| Blackbutt | Rudosols | 120.63 | -30.2 | 311.2 | 19.0 | 145.6 | 5.7 | 5 |
| Blue Mountains | Rudosols | 150.28 | -33.63 | 1165.8 | 12.0 | 324.1 | 4.2 | 11 |
| Bothwell | Sodosols | 147.05 | -42.39 | 546.3 | 10.2 | 102.9 | 3.2 | 14 |
| Boulia | Sodosols | 139.5 | -22.59 | 266.5 | 24.5 | 198.2 | 5.6 | 2 |
| Callitris | Sodosols | 120.71 | -30.18 | 301.3 | 19.2 | 131.1 | 5.4 | 8 |
| Calperum mallee | Sodosols | 140.59 | -34.0 | 260.1 | 17.3 | 185.6 | 7.6 | 10 |
| Cape Tribulation | Tenosols | 145.46 | -16.08 | 3410.1 | 25.6 | 407.0 | 4.2 | 22 |
| Cardwell | Tenosols | 146.13 | -18.44 | 2162.3 | 23.0 | 132.0 | 4.8 | 17 |
| Castlereagh | Tenosols | 150.7 | -32.65 | 772.3 | 15.8 | 205.0 | 5.1 | 6 |
| Claraville | Tenosols | 141.82 | -18.71 | 657.2 | 27.2 | 40.0 | 5.0 | 16 |
| Cocoparra | Tenosols | 146.2 | -34.13 | 437.7 | 16.9 | 213.4 | 5.6 | 2 |
| Cumberland Plain | Tenosols | 150.74 | -33.62 | 773.5 | 17.8 | 113.0 | 4.2 | 12 |
| Curtain Fig | Tenosols | 145.57 | -17.28 | 1410.3 | 21.0 | 3790.0 | 6.5 | 6 |
| Daintree | Tenosols | 145.45 | -16.1 | 3581.0 | 24.6 | 310.8 | 4.8 | 23 |
| Daly river | Tenosols | 131.23 | -14.09 | 1179.9 | 27.5 | 154.7 | 5.3 | 2 |
| Danbulla | Tenosols | 145.6 | -17.11 | 2315.4 | 21.0 | 200.7 | 4.7 | 6 |
| Dorrigo | Tenosols | 152.71 | -30.32 | 1860.6 | 14.8 | 2126.0 | 4.6 | 13 |
| Downfall Creek | Chromosols | 145.59 | -17.15 | 1737.4 | 21.2 | 180.0 | 5.3 | 11 |
| Dry creek | Dermosols | 132.22 | -15.15 | 945.9 | 27.2 | 237.7 | 5.3 | 2 |
| DryMonsoonForest | Dermosols | 130.9 | -12.4 | 1785.1 | 27.3 | 130.2 | 4.9 | 6 |
| Ex-credo | Dermosols | 120.64 | -30.19 | 309.1 | 19.0 | 139.3 | 5.8 | 5 |
| Gnangara-Moore | Dermosols | 115.71 | -31.37 | 648.5 | 18.5 | 135.5 | 5.1 | 30 |
| Great Western Woodlands | Dermosols | 120.69 | -30.26 | 304.5 | 18.9 | 167.0 | 7.9 | 9 |
| Homestead | Kandosols | 120.83 | -30.47 | 291.8 | 19.0 | 401.6 | 6.2 | 10 |
| Howard Springs | Kandosols | 131.11 | -12.45 | 1753.7 | 27.5 | 384.3 | 9.1 | 17 |
| Hunter Valley | Kandosols | 151.17 | -32.57 | 676.5 | 18.0 | 194.8 | 8.9 | 11 |
| Jurien Bay Site 0, Stage1 | Kandosols | 115.08 | -30.4 | 545.2 | 19.2 | 28.8 | 6.9 | 9 |
| Jurien Bay Site 2, Stage3 | Kandosols | 115 | -30.03 | 562.0 | 19.6 | 435.8 | 4.6 | 9 |
| Jurien Bay Site 3, Stage4 | Kandosols | 115.06 | -30.14 | 540.6 | 19.3 | 168.1 | 6.1 | 10 |
| Kaaru Creek | Kandosols | 145.72 | -17.65 | 3091.0 | 21.4 | 168.1 | 6.1 | 13 |
| Kidman Springs Grey Soil | Kandosols | 130.95 | -16.12 | 783.5 | 27.4 | 171.4 | 3.9 | 7 |
| Kidman Springs Red Soil | Kandosols | 130.95 | -16.12 | 783.5 | 27.4 | 92.1 | 3.0 | 7 |
| Ku-ring-gai | Kandosols | 151.15 | -33.68 | 1187.5 | 17.4 | 944.4 | 4.6 | 18 |
| Longley | Kandosols | 147.19 | -42.98 | 989.0 | 11.0 | 1188.6 | 5.0 | 16 |
| Maleny | Kandosols | 152.88 | -26.78 | 1805.7 | 19.5 | 285.5 | 4.3 | 3 |
| Mount Bellenden Ker | Kandosols | 145.85 | -17.26 | 4389.8 | 20.0 | 520.0 | 4.8 | 6 |
| Mount Haig | Kandosols | 145.59 | -17.09 | 2533.9 | 20.4 | 169.2 | 5.0 | 19 |
| Mount Keira | Kandosols | 150.85 | -34.4 | 1522.1 | 16.7 | 156.7 | 4.8 | 27 |
| Macquarie Univ forest | Kandosols | 151.11 | -33.77 | 1086.5 | 17.7 | 245.7 | 4.3 | 3 |
| Oliver Creek | Kurosols | 145.44 | -16.14 | 3340.5 | 24.2 | 510.6 | 5.1 | 11 |
| OpenForest | Kurosols | 131 | -12.7 | 1693.1 | 27.6 | 294.6 | 4.3 | 9 |
| Princess Hills | Kurosols | 145.49 | -18.3 | 878.4 | 21.2 | 270.0 | 4.4 | 17 |
| Pymble | Kurosols | 151.14 | -33.75 | 1142.9 | 17.6 | 314.8 | 4.6 | 16 |
| Royal Nat’l Park Bola Creek | Kurosols | 151.03 | -34.15 | 1137.3 | 17.0 | 143.6 | 5.0 | 25 |
| Royal Nat’l Park Tall Timbers | Hydrosols | 151.11 | -34.1 | 1169.5 | 17.6 | 149.5 | 6.5 | 12 |
| Robson Creek | Hydrosols | 145.63 | -17.12 | 1762.0 | 21.1 | 169.2 | 5.0 | 31 |
| Scheyville | Ferrosols | 150.88 | -33.61 | 845.4 | 17.7 | 236.2 | 4.4 | 12 |
| SolarVillage | Ferrosols | 131.17 | -12.62 | 1661.1 | 27.5 | 287.6 | 4.4 | 2 |
| Sturt Plains | Ferrosols | 133.19 | -17.07 | 637.7 | 26.6 | 288.0 | 4.3 | 2 |
| Swamp | Ferrosols | 131 | -12.7 | 1693.1 | 27.6 | 150.8 | 5.9 | 3 |
| Sydney hiP | Dermosols | 151.27 | -33.6 | 1314.5 | 17.5 | 236.3 | 5.1 | 21 |
| Sydney loP | Dermosols | 151.14 | -33.69 | 1189.1 | 17.4 | 135.7 | 6.0 | 22 |
| Sydney | Ferrosols | 151.1 | -33.95 | 1044.4 | 17.5 | 139.3 | 5.8 | 4 |
| Tower | Calcarosols | 120.65 | -30.19 | 305.3 | 19.1 | 1787.0 | 4.8 | 16 |
| Transcontinentalis | Calcarosols | 120.64 | -30.19 | 309.1 | 19.0 | 302.7 | 3.7 | 7 |
| Tully Falls | Calcarosols | 145.53 | -17.68 | 1612.2 | 19.5 | 237.7 | 5.9 | 9 |
| Warra | Calcarosols | 146.65 | -43.1 | 1597.7 | 11.3 | 260.0 | 6.2 | 14 |
| WNSW hi P | Kandosols | 146.15 | -32.97 | 384.1 | 18.1 | 310.0 | 4.5 | 23 |
| WNSW lo P | Kandosols | 146.15 | -32.98 | 384.1 | 18.1 | 610.8 | 5.0 | 21 |
| Woodland | Vertosols | 131 | -12.7 | 1693.1 | 27.6 | 169.2 | 5.0 | 8 |
| Yengo | Organosols | 150.92 | -32.78 | 794.6 | 16.7 | 222.9 | 4.4 | 15 |

**Table S2** Source publications. Citation information for published datasets included in the present study. For cases where data were part of a larger data compilation, we cite the original publication(s) as well as the data compilation study.

Atkin, O.K., Bloomfield, K.J., Reich, P.B., Tjoelker, M.G., Asner, G.P., Bonal, D., Bönisch, G., Bradford, M.G., Cernusak, L.A., Cosio, E.G., & Creek, D. (2015). Global variability in leaf respiration in relation to climate, plant functional types and leaf traits. *New Phytologist*, *206*(2), 614-636.

Bauman D., Fortunel C., Cernusak L.A., Bentley L.P., McMahon S.M., Rifai S.W., Aguirre-Gutiérrez J., Oliveras I., Bradford M., Laurance S.G.W. et al. (2022). Tropical tree growth sensitivity to climate is driven by species intrinsic growth rate and leaf traits. Global Change Biology, In press: https://doi.org/10.1111/gcb.15982.

Bloomfield, K.J., Cernusak, L.A., Eamus, D., Ellsworth, D.S., Colin Prentice, I., Wright, I.J., Boer, M.M., Bradford, M.G., Cale, P., Cleverly, J., & Egerton, J.J. (2018). A continental‐scale assessment of variability in leaf traits: Within species, across sites and between seasons. *Functional Ecology*, *32*(6), 1492-1506.

Cernusak, L.A., Hutley, L.B., Beringer, J., & Tapper, N.J., 2006. Stem and leaf gas exchange and their responses to fire in a north Australian tropical savanna. *Plant, Cell & Environment*, *29*(4), pp.632-646.

Cernusak, L.A., Hutley, L.B., Beringer, J., Holtum, J.A., & Turner, B.L. (2011). Photosynthetic physiology of eucalypts along a sub-continental rainfall gradient in northern Australia. *Agricultural and Forest Meteorology*, *151*(11), 1462-1470.

Eamus, D., Myers, B., Duff, G., & Williams, R. (2000). A cost-benefit analysis of leaves of eight Australian savanna tree species of differing leaf life-span. *Photosynthetica*, *36*(4), 575-586.

Eamus, D., & Prichard, H. (1998). A cost-benefit analysis of leaves of four Australian savanna species. *Tree Physiology*, *18*(8-9), 537-545.

Fürstenau Togashi, H, Bloomfield, K., Prentice, C., Evans, B., Atkin, O., Prober, S., & Macfarlane, C. (2015): Leaf Level Physiology, Chemistry and Structural Traits, Great Western Woodlands SuperSite, 2014. TERN Australian SuperSite Leaf Level Physiology, Chemistry and Structural Traits, Great Western Woodlands SuperSite, 2014 Network. http://supersites.tern.org.au/knb/metacat/supersite.302.3/html. Accessed on 4/24/2019.

Gleason, S.M., Butler, D.W., Ziemińska, K., Waryszak, P., & Westoby, M. (2012). Stem xylem conductivity is key to plant water balance across Australian angiosperm species. *Functional Ecology*, 26(2), 343-352.

Gleason, S.M., Butler, D.W., & Waryszak, P. (2013). Shifts in leaf and stem hydraulic traits across aridity gradients in eastern Australia. *International Journal of Plant Sciences*, *174*(9), 1292-1301.

Grassi, G., Meir, P., Cromer, R., Tompkins, D., & Jarvis, P.G. (2002). Photosynthetic parameters in seedlings of Eucalyptus grandis as affected by rate of nitrogen supply. *Plant, Cell & Environment*, *25*,1677-1688.

Gray, E. F., Wright, I.J., Falster, D.S., Eller, A.S., Lehmann, C.E.R., Bradford, M.G., & Cernusak, L.A. (2019). Leaf: wood allometry and functional traits together explain substantial growth rate variation in rainforest trees. *AoB Plants,* *11*(3), plz024.

Guilherme Pereira, C., Hayes, P. E., O’Sullivan, O. S., Weerasinghe, L. K., Clode, P. L., Atkin, O. K., & Lambers, H. (2019). Trait convergence in photosynthetic nutrient‐use efficiency along a 2‐million year dune chronosequence in a global biodiversity hotspot. *Journal of Ecology*, *107*(4), 2006-2023.

Han, Q., Kawasaki, T., Nakano, T., & Chiba, Y. (2008). Leaf-age effects on seasonal variability in photosyntheticparameters and its relationships with leaf mass per area andleaf nitrogen concentration within a Pinus densiﬂora crown. *Tree Physiology*, *28*, 551-558.

Leishman, M.R., Thomson, V.P., & Cooke, J. (2010). Native and exotic invasive plants have fundamentally similar carbon capture strategies. *Journal of Ecology*, *98*(1), 28-42.

Maire, V., Wright, I.J., Prentice, I.C., Batjes, N.H., Bhaskar, R., Van Bodegom, P.M., Cornwell, W.K., Ellsworth, D., Niinemets, Ü., Ordonez, A., & Reich, P.B. (2015). Global effects of soil and climate on leaf photosynthetic traits and rates. *Global Ecology and Biogeography*, *24*(6), 706-717.

McPherson, S., Eamus, D., & Murray, B.R. (2004). Seasonal impacts of leaf atributes of several tree species growing in three diverse ecosystems in south-eastern Australia. *Australian Journal of Botany*, *52*, 293-301.

Niinemets, Ü., Wright, I.J., & Evans, J.R. (2009). Leaf mesophyll diffusion conductance in 35 Australian sclerophylls covering a broad range of foliage structural and physiological variation. *Journal of Experimental Botany*, *60*(8), 2433-2449.

Nolan, R.H., Fairweather, K.A., Tarin, T., Santini, N.S., Cleverly, J., Faux, R., & Eamus, D. (2017). Divergence in plant water-use strategies in semiarid woody species. *Functional Plant Biology*, *44*(11), 1134-1146

Nolan, R.H., Tarin, T., Fairweather, K.A., Cleverly, J., & Eamus, D. (2017). Variation in photosynthetic traits related to access to water in semiarid Australian woody species. *Functional Plant Biology*, *44*(11), 1087-1097

Prior, L. D., Eamus, D., & Bowman, D. M. (2003). Leaf attributes in the seasonally dry tropics: a comparison of four habitats in northern Australia. *Functional Ecology*, 504-515.

Richards, A. E., & Schmidt, S. (2010). Complementary resource use by tree species in a rain forest tree plantation. *Ecological Applications*, *20*(5), 1237-1254.

Walker, A.P., I. Aranda, A.P. Beckerman, H. Bown, L.A. Cernusak, Q.L. Dang, T.F. Domingues, L. Gu, S. Guo, Q. Han, J. Kattge, M. Kubiske, D. Manter, E. Merilo, G. Midgley, A. Porte, J.C. Scales, D. Tissue, T. Turnbull, C. Warren, G. Wohlfahrt, F.I. Woodward, & S.D. Wullschleger. (2014). A Global Data Set of Leaf Photosynthetic Rates, Leaf N and P, and Specific Leaf Area. Data set. Available on-line [http://daac.ornl.gov] from Oak Ridge National Laboratory Distributed Active Archive Center, Oak Ridge, Tennessee, USA. http://dx.doi.org/10.3334/ORNLDAAC/1224

Walker, A.P., Beckerman, A.P., Gu, L., Kattge, J., Cernusak, L.A., Domingues, T.F., Scales, J.C., Wohlfahrt, G., Wullschleger, S.D., & Woodward, F.I. (2014). The relationship of leaf photosynthetic traits - V cmax and J max - to leaf nitrogen, leaf phosphorus, and specific leaf area: a meta-analysis and modeling study. *Ecology and Evolution*, *4*(16), 3218-3235.

Warren, C.R., & Adams, M.A. (2001). Distribution of N, Rubisco and photosynthesis in Pinus pinaster and acclimation to light. *Plant, Cell & Environment*, *24*(6), 597-609.

Weerasinghe, L.K., Creek, D., Crous, K.Y., Xiang, S., Liddell, M.J., Turnbull, M.H., & Atkin O.K. (2014). Canopy position affects the relationships between leaf respiration and associated traits in a tropical rainforest in Far North Queensland. *Tree Physiology*, *34*(6), 564-584.

Wright, I. J., Reich, P. B., & Westoby, M. (2001). Strategy shifts in leaf physiology, structure and nutrient content between species of high‐and low‐rainfall and high‐and low‐nutrient habitats. *Functional Ecology*, *15*(4), 423-434.

Wright, I. J., Cooke, J., Cernusak, L.A., Hutley, L.B., Scalon, M.C., Tozer, W.C., & Lehmann, C.E. (2019). Stem diameter growth rates in a fire‐prone savanna correlate with photosynthetic rate and branch‐scale biomass allocation, but not specific leaf area. *Austral Ecology*, *44*(2), 339-350.

**Table S3**. Focal traits and abiotic variables. See Xu and Hutchinson (2011)^1^ for additional details regarding methodology.

|  | **Abbreviation** | **Definition** | **Units** |
| --- | --- | --- | --- |
|  | C_i_:C_a_ | CO_2_ drawdown, internal [CO_2_]/ambient [CO_2_] | none |
|  | g_sw_ | Stomatal conductance to water vapor | mmol m^-2^ s^-1^ |
|  | V_cmax 25_ | Carboxylation capacity at a leaf temperature of 25°C | µmol m^-2^ s^-1^ |
|  | N_area_ | leaf nitrogen concentration per unit leaf area | g m^-2^ |
| Climate | MAP | Mean annual precipitation | mm |
|  | MAT | Mean annual temperature | °C |
|  | MI | Moisture index (MAP/PET) | none |
|  | AET | Actual evapotranspiration | mm month^-1^ |
|  | PET | Potential evapotranspiration | mm month^-1^ |
|  | Radiation | Solar radiation | MJ m^-2^ yr^-1^ |
|  | GDD_0_ | Growing degree days (number of days with air temperature above 0°C) | days |
|  | Isothermality | (Annual mean diurnal range - Temperature annual range) × 100 | none |
|  | Max Temp | Annual mean of daily maximum temperature | °C |
|  | Min Temp | Annual mean of daily minimum temperature | °C |
|  | VPD | Vapor pressure deficit at 9 am | kPa |
|  | Temp Seasonality | Standard deviation of temperature × 100 | none |
| Soil | Soil Phosphorus | Total soil phosphorus concentration | g kg^-1^ |
|  | pH | pH | none |
|  | Soil Nitrogen | Total soil nitrogen concentration | g kg^-1^ |
|  | ECE | Effective cation exchange capacity | meq 100 g^-1^ |
|  | BDW | Bulk density whole earth | g cm^-3^ |
|  | SOC | Soil organic carbon concentration | % |
|  | %Clay | % soil composed of clay | % |
|  | %Silt | % soil composed of silt | % |
|  | %Sand | % soil composed of sand | % |

^1^ Xu, T. and Hutchinson, M., 2011. ANUCLIM version 6.1 user guide. The Australian National University, Fenner School of Environment and Society, Canberra, 90.

**Fig. S2** Climate-soil space encompassed by 67 study sites (grey circles), distributed across the Australian continent. R^2^ values and trendlines are shown for all relationships. All axes are log_10_-scaled.

**
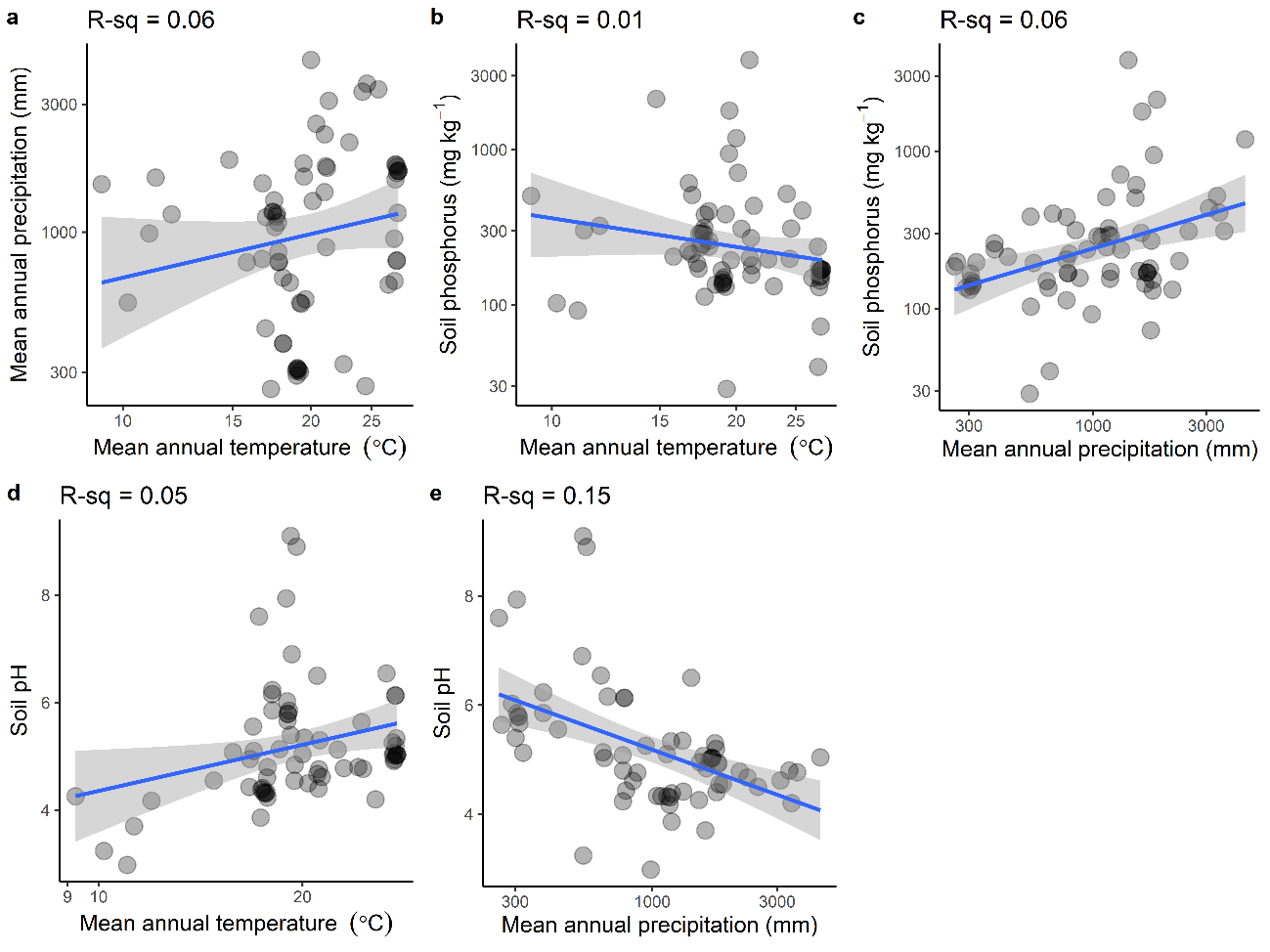
**

**Fig. S3.** Correlation matrix of climate and soil variables. For this analysis, all variables were log10-transformed to control for significant differences in scale and variation.

**
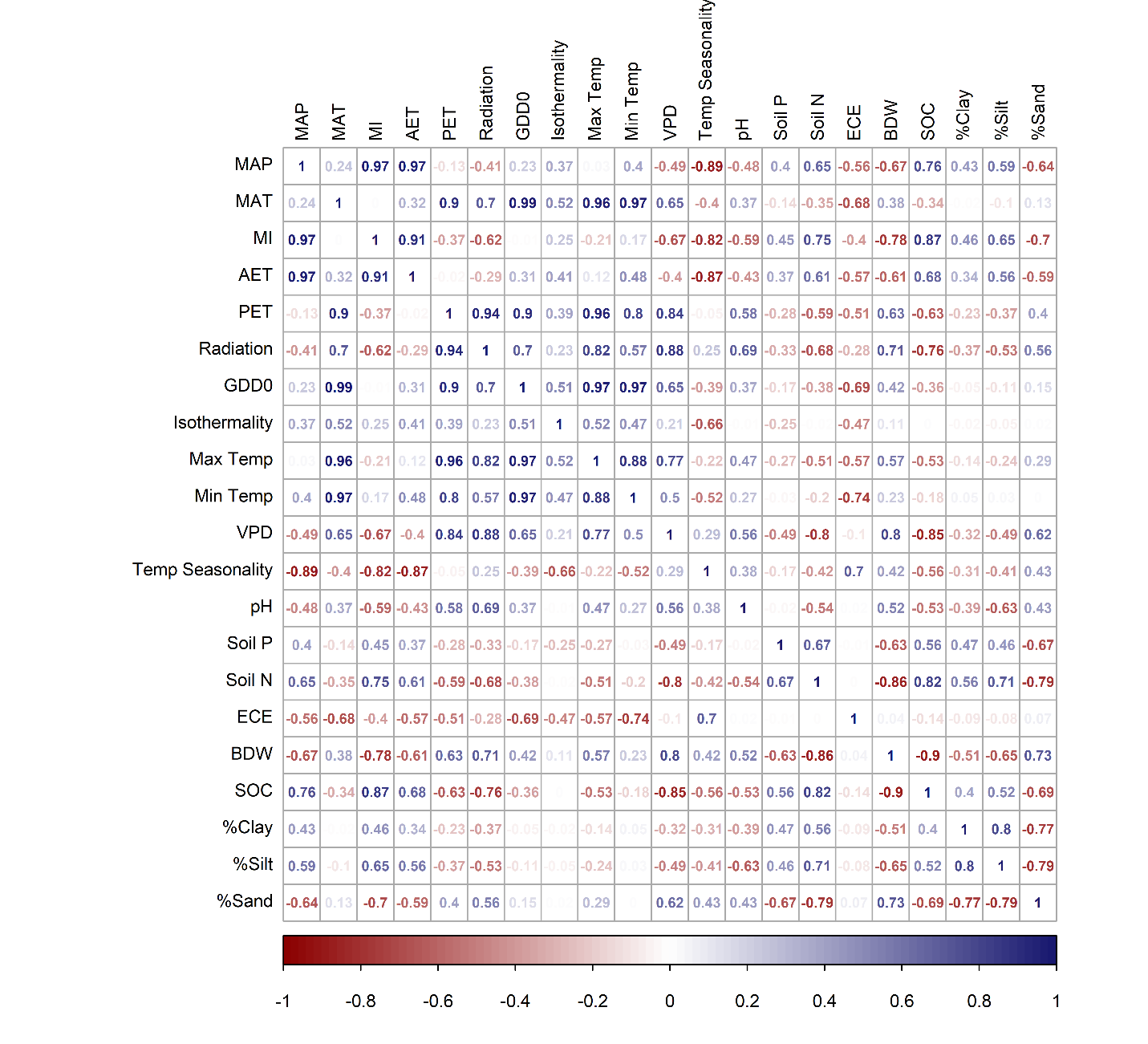
**

**Fig. S4** Leaf nitrogen concentrations for nitrogen-fixing and non-fixing species. Differences in a) mass- and b) area-based leaf nitrogen (N) concentration for N-fixing (N=132) and non-N-fixing species (N=629).
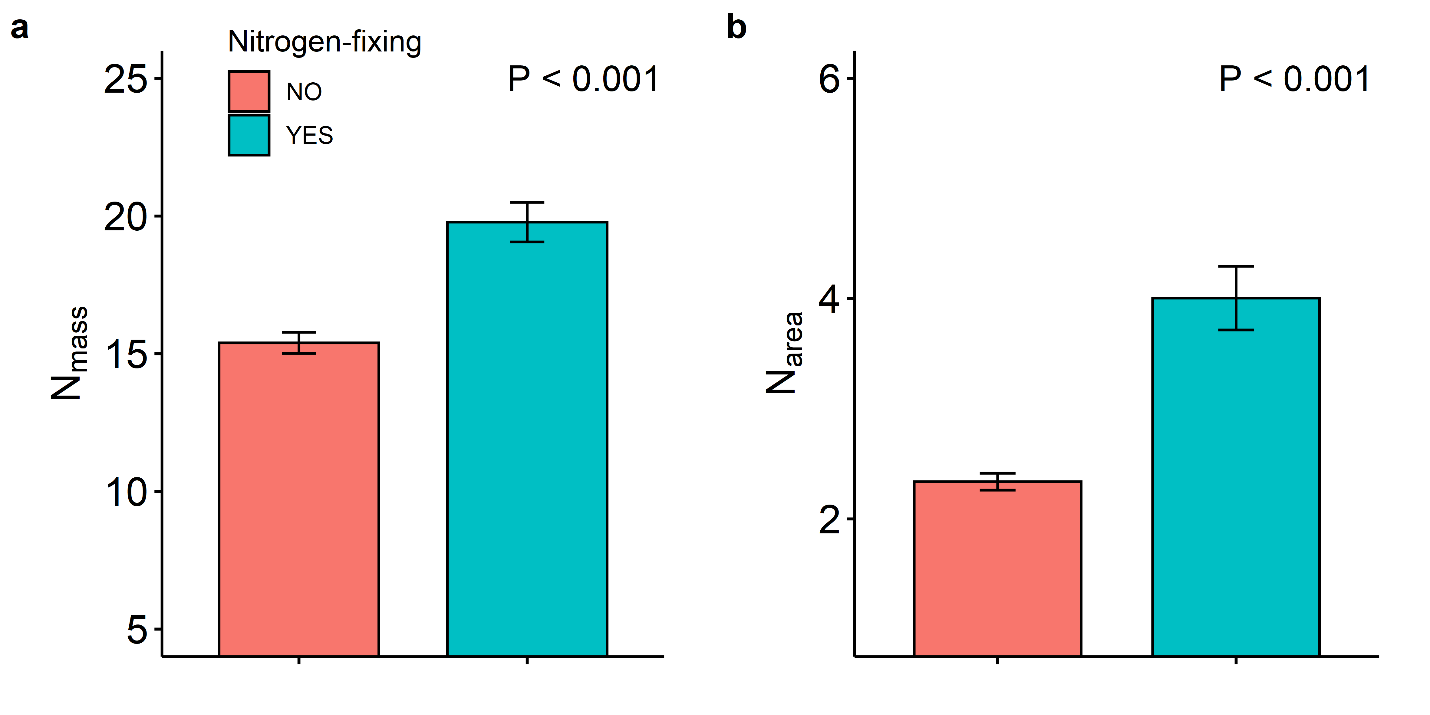


**Fig. S5** Partial regression plots of soil and climate effects on a-d) C_i_:C_a_ and on the slope relationship between e-h) leaf nitrogen concentration (N) on an area basis, N_area_, and stomatal conductance, g_sw_ and i-l) photosynthetic carboxylation, V_cmax 25_ and g_sw_. Nitrogen-fixing species have been excluded from this analysis. Points represent species site means and trendlines are shown for statistically significant relationships, where solid lines indicate *P* < 0.05 and dashed lines represent marginally significant results (0.05 < *P* < 0.10). *P*-values above each panel indicate the statistical significance of each variable in as multiple regression. Higher β values indicate a stronger effect size, where β values are the regression weights for standardized variables and represent the change in the slope value (in standard deviations) associated with a change of one standard deviation in a predictor while holding constant the value(s) of the other predictor(s). Abbreviations: Soil total phosphorus (Soil P, mg kg^-1^) concentration, Mean annual precipitation (MAP, mm), Mean annual temperature (MAT, °C). Note the axes are logarithmically scaled.


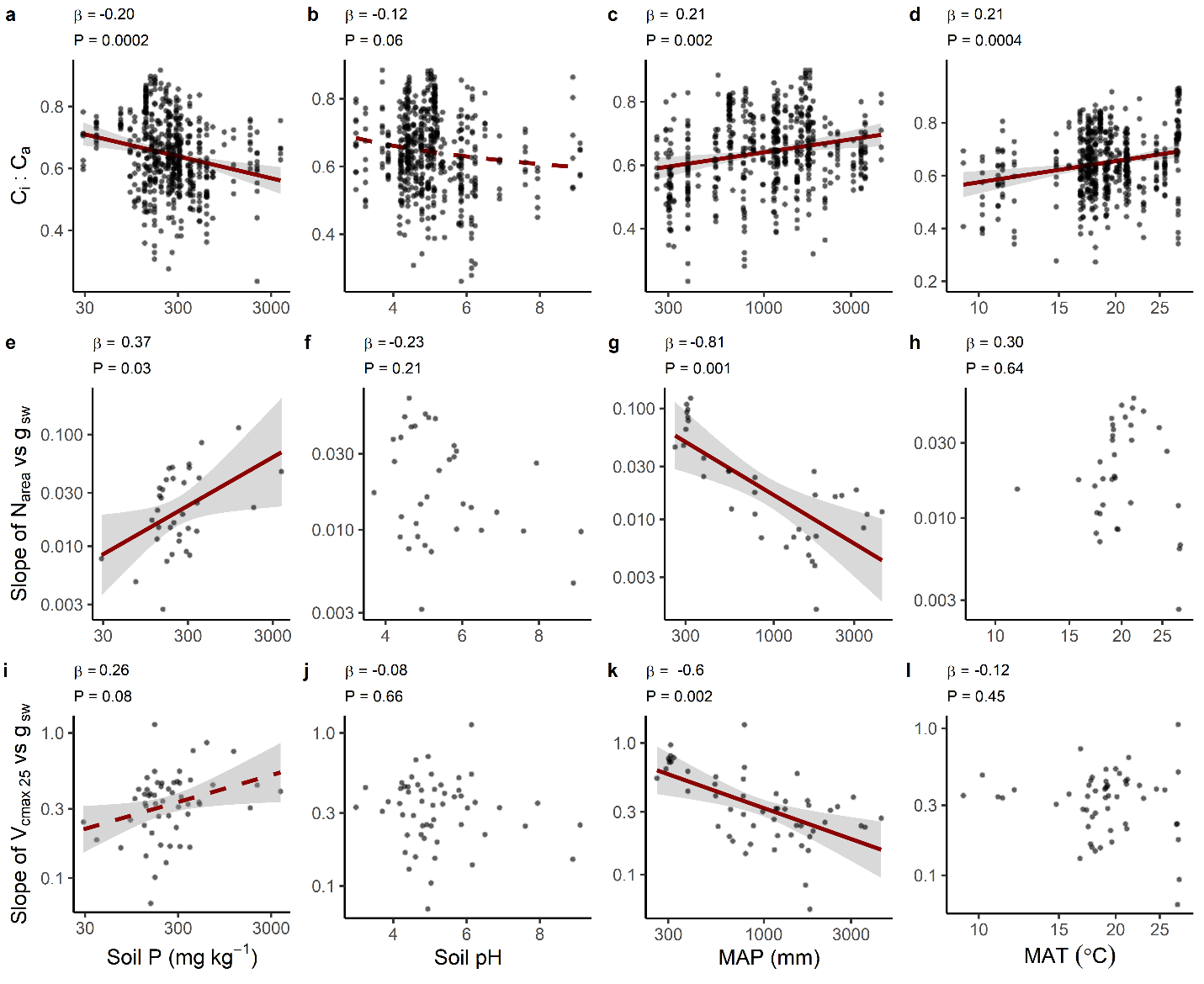


**Table S4.** Trait × environment relationships using ordinary least squares linear regression analysis. With the exception of soil pH, all abiotic variables have been log_10_ transformed to meet normality assumptions. Values shown below are slope coefficients (S) and R^2^ values. Italics indicates *P* > 0.05. Abbreviations follow Table S1. Note that the N_area_-g_sw_ and V_cmax 25_-g_sw_ relationships were evaluated using site level data.

|  | **Soil P** | | **pH** | | **Soil N** | | **ECE** | | **BDW** | | **SOC** | | **Clay** | | **Silt** | | **Sand** | | |
| --- | --- | --- | --- | --- | --- | --- | --- | --- | --- | --- | --- | --- | --- | --- | --- | --- | --- | --- | --- |
|  | **S** | **R^2^** | **S** | **R^2^** | **S** | **R^2^** | **S** | **R^2^** | **S** | **R^2^** | **S** | **R^2^** | **S** | **R^2^** | **S** | **R^2^** | **S** | **R^2^** |  |
| N_area_-g_sw_ | *0.07* | *-0.02* | *0.07* | *0.01* | *-0.28* | *0.02* | 0.96 | 0.24 | *0.85* | *<0.01* | *-0.4* | *0.05* | *0.21* | *<0.01* | *-0.05* | *<0.01* | *-0.07* | *<0.01* |  |
| V_cmax 25_-g_sw_ | *0.04* | *-0.02* | *0.05* | *0.02* | *-0.12* | *0.004* | 0.58 | 0.23 | *0.16* | *<0.01* | *-0.15* | *0.009* | *0.13* | *<0.01* | *-0.05* | *<0.01* | *-0.12* | *<0.01* |  |
| C_i_:C_a_ | -0.03 | 0.01 | -0.01 | 0.03 | *0.02* | *0.003* | -0.02 | 0.08 | *0.03* | *<0.01* | -0.07 | 0.01 | -0.003 | 0.03 | *0.02* | *<0.01* | *0.003* | *<0.01* |  |
| g_sw_ | -0.37 | 0.13 | *0.0004* | *<0.01* | -0.25 | 0.05 | 2.30 | 0.06 | -0.24 | 0.10 | -0.59 | 0.04 | -0.27 | 0.14 | 0.97 | 0.07 | 0.12 | 0.11 |  |
| V_cmax 25_ | -0.28 | 0.16 | 0.45 | 0.03 | -0.31 | 0.17 | *2.20* | *<0.01* | -0.33 | 0.21 | -0.38 | 0.15 | -0.27 | 0.12 | 0.88 | 0.14 | 0.11 | 0.19 |  |
| N_area_ | -0.11 | 0.02 | 1.10 | 0.13 | -0.35 | 0.24 | 2.10 | 0.12 | -0.37 | 0.19 | -0.05 | 0.23 | *-0.20* | *<0.01* | 0.61 | 0.09 | 0.07 | 0.12 |  |
| P_area_ | *0.02* | *<0.01* | 0.09 | 0.19 | -0.28 | 0.14 | 0.11 | 0.01 | 1.6 | 0.10 | -0.28 | 0.13 | *-0.02* | *<0.01* | -0.26 | 0.12 | 0.53 | 0.07 |  |
| A_area_ | -0.31 | 0.19 | 0.03 | 0.01 | -0.28 | 0.13 | -0.14 | 0.02 | 2.1 | 0.18 | -0.27 | 0.10 | -0.46 | 0.17 | -0.27 | 0.13 | 0.87 | 0.18 |  |
| LMA | -0.18 | 0.07 | 0.60 | 0.05 | -0.26 | 0.13 | 0.25 | 0.08 | 1.5 | 0.11 | -0.29 | 0.15 | -0.21 | 0.05 | -0.21 | 0.10 | 0.67 | 0.13 |  |
| PNUE | -0.12 | 0.02 | -0.04 | 0.02 | 0.16 | 0.03 | -0.43 | 0.12 | -0.71 | 0.01 | 0.25 | 0.06 | -0.32 | 0.07 | *-0.01* | *<0.01* | *0.07* | *<0.01* |  |
| PPUE | -0.34 | 0.12 | -0.07 | 0.06 | *0.02* | *<0.001* | -0.21 | 0.03 | *0.37* | *<0.01* | *0.05* | *<0.01* | -0.45 | 0.10 | *0.05* | *<0.01* | 0.31 | 0.01 |  |

|  | **MAP** | | **MAT** | | **MI** | | **AET** | | **PET** | | **Radiation** | | **GDD_0_** | | |
| --- | --- | --- | --- | --- | --- | --- | --- | --- | --- | --- | --- | --- | --- | --- | --- |
|  | **S** | **R^2^** | **S** | **R^2^** | **S** | **R^2^** | **S** | **R^2^** | **S** | **R^2^** | **S** | **R^2^** | **S** | **R^2^** |  |
| N_area_-g_sw_ | -0.72 | 0.32 | -3.2 | 0.28 | -0.59 | 0.23 | -1.6 | 0.43 | *-1.9* | *0.06* | *-0.76* | *<0.01* | -3.1 | 0.27 |  |
| V_cmax 25_-g_sw_ | -0.45 | 0.23 | -1.0 | 0.12 | -0.34 | 0.14 | -0.99 | 0.29 | *-0.84* | *0.05* | *-0.62* | *<0.01* | -1.0 | 0.12 |  |
| C_i_:C_a_ | 0.08 | 0.07 | 0.18 | 0.04 | 0.06 | 0.05 | 0.17 | 0.09 | 0.12 | 0.01 | *0.04* | *<0.01* | 0.18 | 0.04 |  |
| g_sw_ | *0.02* | *<0.01* | 0.96 | 0.07 | *-0.07* | *<0.01* | *0.14* | *<0.01* | 1.30 | 0.10 | 1.90 | 0.08 | 1.00 | 0.08 |  |
| V_cmax 25_ | -0.26 | 0.11 | *0.18* | *<0.01* | -0.27 | 0.14 | -0.49 | 0.10 | 0.69 | 0.06 | 1.50 | 0.09 | 0.24 | 0.01 |  |
| N_area_ | -0.39 | 0.38 | -0.63 | 0.05 | -0.37 | 0.36 | -0.86 | 0.45 | *0.25* | *<0.01* | 1.30 | 0.07 | -0.61 | 0.04 |  |
| P_area_ | -0.31 | 0.07 | *0.15* | *0.04* | -0.31 | 0.05 | -0.64 | 0.09 | 0.64 | 0.01 | 1.5 | <0.01 | *0.10* | *0.04* |  |
| A_area_ | -0.12 | <0.01 | 0.52 | 0.07 | -0.17 | <0.01 | -0.19 | <0.01 | 0.90 | 0.10 | 1.5 | 0.08 | 0.57 | 0.08 |  |
| LMA | -0.34 | 0.11 | -0.27 | <0.01 | -0.33 | 0.14 | -0.69 | 0.10 | 0.25 | 0.06 | 1.1 | 0.09 | -0.26 | 0.01 |  |
| PNUE | 0.36 | 0.38 | 1.2 | 0.05 | 0.31 | 0.36 | 0.82 | 0.45 | 0.62 | <0.01 | *0.05* | *0.07* | 1.2 | 0.04 |  |
| PPUE | 0.22 | 0.18 | 0.34 | <0.01 | 0.18 | 0.21 | 0.53 | 0.21 | *0.16* | *0.05* | *-0.18* | *0.11* | 0.43 | <0.01 |  |

|  | **Isothermality** | | **Max Temp** | | **Min Temp** | | **VPD** | | **Temp Seasonality** | |  |
| --- | --- | --- | --- | --- | --- | --- | --- | --- | --- | --- | --- |
|  | **S** | **R^2^** | **S** | **R^2^** | **S** | **R^2^** | **S** | **R^2^** | **S** | **R^2^** | |
| N_area_-g_sw_ | -8.5 | 0.64 | -3.3 | 0.18 | -2.2 | 0.33 | *-0.02* | *<0.01* | 2.3 | 0.54 | |
| V_cmax 25_-g_sw_ | -4.5 | 0.40 | -1.1 | 0.09 | -0.7 | 0.14 | *-0.06* | *<0.01* | 1.4 | 0.41 | |
| C_i_:C_a_ | 0.86 | 0.12 | 0.18 | 0.02 | 0.13 | 0.05 | *-0.003* | *<0.01* | -0.26 | 0.12 | |
| g_sw_ | 3.70 | 0.13 | 1.40 | 0.10 | 0.50 | 0.04 | 0.45 | 0.06 | -0.42 | 0.02 | |
| V_cmax 25_ | *0.24* | *<0.01* | 0.56 | 0.03 | *-0.03* | *<0.01* | 0.42 | 0.12 | 0.50 | 0.06 | |
| N_area_ | -2.60 | 0.21 | *-0.23* | *<0.01* | -0.65 | 0.12 | 0.49 | 0.15 | 0.96 | 0.37 | |
| P_area_ | -1.0 | 0.12 | 0.45 | 0.02 | *-0.07* | *0.05* | 0.46 | <0.01 | 0.69 | 0.12 | |
| A_area_ | 1.4 | 0.13 | 0.88 | 0.10 | 0.22 | 0.04 | 0.39 | 0.06 | *0.06* | *0.02* | |
| LMA | -1.5 | <0.01 | *-0.04* | *0.03* | -0.31 | <0.01 | 0.36 | 0.09 | 0.85 | 0.06 | |
| PNUE | 3.4 | 0.21 | 1.1 | <0.01 | 0.93 | 0.12 | *-0.12* | *0.15* | -1.0 | 0.37 | |
| PPUE | 2.3 | 0.02 | 0.37 | 0.02 | 0.28 | <0.01 | *-0.08* | *0.14* | -0.67 | 0.14 | |

**Table S5.** Regression weights for standardized variables (i.e., β values) from a multiple regression and associated *P*-values. Column names in each table represent the predictors included in the model. R^2^ values are shown for each model. Grey font indicates a lack of statistical significance at α = 0.10. Response and predictor variables have been log_10_ transformed to meet normality assumptions. Abbreviations follow Table S1.

|  | **Soil P** | | **pH** | | **MAP** | | **MAT** | | **R^2^** |
| --- | --- | --- | --- | --- | --- | --- | --- | --- | --- |
|  | **β** | ***P*** | **β** | ***P*** | **β** | ***P*** | **β** | ***P*** |  |
| C_i_:C_a_ | -0.21 | <0.001 | -0.10 | 0.10 | 0.26 | <0.001 | 0.18 | 0.001 | 0.14 |
| g_sw_ | -0.39 | <0.001 | -0.02 | 0.70 | 0.12 | 0.04 | 0.20 | <0.001 | 0.20 |
| V_cmax 25_ | -0.30 | <0.001 | 0.02 | 0.74 | -0.22 | <0.001 | 0.08 | 0.14 | 0.21 |
| N_area_ | 0.14 | 0.002 | -0.07 | 0.19 | -0.76 | <0.001 | 0.10 | 0.04 | 0.40 |
| P_area_ | 0.23 | <0.001 | 0.15 | 0.02 | -0.50 | <0.001 | 0.16 | 0.003 | 0.29 |
| A_area_ | -0.41 | <0.001 | 0.03 | 0.62 | -0.01 | 0.81 | 0.14 | 0.007 | 0.22 |
| LMA | -0.06 | 0.18 | -0.02 | 0.68 | -0.50 | <0.001 | 0.05 | 0.31 | 0.25 |
| PNUE | -0.35 | <0.001 | 0.25 | <0.001 | 0.74 | <0.001 | -0.03 | 0.62 | 0.32 |
| PPUE | -0.47 | <0.001 | -0.10 | 0.12 | 0.34 | <0.001 | 0.02 | 0.69 | 0.27 |

|  | **Soil P** | | **pH** | | **Soil N** | | **MAP** | | **MAT** | | **VPD** | | **Radiation** | | **R^2^** |
| --- | --- | --- | --- | --- | --- | --- | --- | --- | --- | --- | --- | --- | --- | --- | --- |
|  | **β** | ***P*** | **β** | ***P*** | **β** | ***P*** | **β** | ***P*** | **β** | ***P*** | **β** | ***P*** | **β** | ***P*** |  |
| C_i_:C_a_ | -0.26 | <0.001 | -0.12 | 0.08 | 0.10 | 0.27 | 0.28 | 0.005 | 0.07 | 0.52 | -0.12 | 0.25 | 0.27 | 0.009 | 0.15 |
| g_sw_ | -0.39 | <0.001 | -0.14 | 0.03 | 0.01 | 0.88 | 0.39 | <0.001 | -0.23 | 0.04 | -0.03 | 0.75 | 0.62 | <0.001 | 0.25 |
| V_cmax 25_ | -0.19 | 0.002 | -0.10 | 0.16 | -0.18 | 0.04 | 0.10 | 0.35 | -0.34 | 0.002 | 0.10 | 0.37 | 0.40 | <0.001 | 0.23 |
| N_area_ | 0.17 | 0.001 | -0.04 | 0.55 | -0.23 | 0.02 | -1.06 | <0.001 | 0.57 | 0.001 | 0.06 | 0.63 | -0.70 | <0.001 | 0.44 |
| P_area_ | 0.31 | <0.001 | 0.17 | 0.01 | -0.18 | 0.04 | -0.39 | 0.01 | 0.16 | 0.37 | 0.28 | 0.009 | -0.34 | 0.04 | 0.32 |
| A_area_ | -0.36 | <0.001 | -0.08 | 0.21 | -0.10 | 0.26 | 0.19 | 0.05 | -0.16 | 0.13 | -0.09 | 0.35 | 0.47 | <0.001 | 0.24 |
| LMA | -0.04 | 0.48 | -0.03 | 0.65 | 0.01 | 0.90 | -0.27 | 0.03 | -0.25 | 0.08 | 0.17 | 0.08 | 0.19 | 0.17 | 0.26 |
| PNUE | -0.31 | <0.001 | 0.27 | <0.001 | 0.14 | 0.23 | 1.55 | <0.001 | -0.95 | <0.001 | 0.41 | <0.001 | 0.72 | <0.001 | 0.37 |
| PPUE | -0.52 | <0.001 | -0.14 | 0.06 | 0.08 | 0.42 | 0.36 | 0.03 | -0.11 | 0.58 | -0.22 | 0.05 | 0.40 | 0.03 | 0.29 |
